# *Ralstonia chuxiongensis* sp. nov., *Ralstonia mojiangensis* sp. nov., and *Ralstonia soli* sp. nov., isolated from tobacco fields, are three novel species in the family *Burkholderiaceae*

**DOI:** 10.3389/fmicb.2023.1179087

**Published:** 2023-05-05

**Authors:** Can-Hua Lu, Ying-Ying Zhang, Ning Jiang, Wei Chen, Xiaoli Shao, Zhi-Ming Zhao, Wen-Lin Lu, Xiaodong Hu, Yi-Xuan Xi, Si-Yuan Zou, Qiu-Ju Wei, Zhong-Long Lin, Li Gong, Xiao-Tong Gai, Li-Qun Zhang, Jun-Ying Li, Yan Jin, Zhen-Yuan Xia

**Affiliations:** ^1^Yunnan Academy of Tobacco Agricultural Sciences, Kunming, China; ^2^College of Plant Protection, China Agricultural University, Beijing, China; ^3^Puer Branch of Yunnan Tobacco Company, Puer, China; ^4^Baoshan Branch of Yunnan Tobacco Company, Baoshan, China; ^5^Zhaotong Branch of Yunnan Tobacco Company, Zhaoyang, China; ^6^Chuxiong Branch of Yunnan Tobacco Company, Chuxiong, China; ^7^China National Tobacco Corporation Yunnan Company, Kunming, China

**Keywords:** *Burkholderiaceae*, *Ralstonia*, sp. nov., phylogeny, taxonomy, average nucleotide identity, digital DNA–DNA hybridization (dDDH)

## Abstract

Eight Gram-negative, aerobic, motile with paired polar flagella and rod-shaped bacteria were isolated from six tobacco fields in Yunnan, PR China. 16S rRNA gene sequence analysis revealed that all the strains belonged to the genus *Ralstonia*. Among them, strain 22TCCZM03-6 had an identical 16S rRNA sequence to that of *R. wenshanensis* 56D2^T^, and the other strains were closely related to *R. pickettii* DSM 6297^T^ (98.34–99.86%), *R. wenshanensis* 56D2^T^ (98.70–99.64%), and *R. insidiosa* CCUG 46789^T^ (97.34–98.56%). Genome sequencing yielded sizes ranging from 5.17 to 5.72 Mb, with overall G + C contents of 63.3–64.1%. Pairwise genome comparisons showed that strain 22TCCZM03-6 shared average nucleotide identity (ANI) and digital DNA–DNA hybridization (dDDH) values above the species cut-off with *R. wenshanensis* 56D2^T^, suggesting that strain 22TCCZM03-6 is a special strain of the *R. wenshanensis*. Five strains, including 21MJYT02-10^T^, 21LDWP02-16, 22TCJT01-1, 22TCCZM01-4, and 22TCJT01-2, had ANI values >95% and dDDH values >70% when compared with each other. These five strains had ANI values of 73.32–94.17% and dDDH of 22.0–55.20% with the type strains of the genus *Ralstonia* individually, supporting these five strains as a novel species in the genus *Ralstonia*. In addition, strains 21YRMH01-3^T^ and 21MJYT02-11^T^ represent two independent species. They both had ANI and dDDH values below the thresholds for species delineation when compared with the type species of the genus *Ralstonia*. In strains 21YRMH01-3^T^ and 21MJYT02-10^T^, the main fatty acids were summed features 3, 8, and C_16:0_; however, strain 21MJYT02-11^T^ contained C_16:0_, cyclo-C_17:0_, and summed features 3 as major fatty acids. The main polar lipids, including diphosphatidylglycerol, phosphatidylglycerol, and phosphatidylethanolamine, were identified from strains 21YRMH01-3^T^, 21MJYT02-10^T^, and 21MJYT02-11^T^. The ubiquinones Q-7 and Q-8 were also detected in these strains, with Q-8 being the predominant quinone. Based on the above data, we propose that the eight strains represent one known species and three novel species in the genus *Ralstonia*, for which the names *Ralstonia chuxiongensis* sp. nov., *Ralstonia mojiangensis* sp. nov., and *Ralstonia soli* sp. nov. are proposed. The type strains are 21YRMH01-3^T^ (=GDMCC 1.3534^T^ = JCM 35818^T^), 21MJYT02-10^T^ (=GDMCC 1.3531^T^ = JCM 35816^T^), and 21MJYT02-11^T^ (=GDMCC 1.3532^T^ = JCM 35817^T^), respectively.

## Introduction

The genus *Ralstonia*, a member of the family *Burkholderiaceae* within the class *Betaproteobacteria*, was named in honor of the American bacteriologist Ericka Ralston (Yabuuchi et al., 1995). According to the List of Prokaryotic names with Standing in Nomenclature^1^ (Parte et al., 2020), the genus *Ralstonia* comprises eight species with validly published names at present, including *R. mannitolilytica*, *R. insidiosa*, *R. pickettii* (the type species), *R. pseudosolanacearum*, *R. solanacearum*, *R. syzygii, R. wenshanensis* and a recently proposed novel species *R. nicotianae* (Safni et al., 2014; Meier-Kolthoff et al., 2022; Lu et al., 2022a; Liu et al., 2023). *R. solanacearum* was initially placed in the genus *Bacillus* and then reclassified multiple times before being named the current species (Yabuuchi et al., 1995; Paudel et al., 2020). *R. solanacearum* strains have been classified into five races based on their pathogenicity toward different hosts (Buddenhagen and Kelman, 1964), three biovars based on a strain’s ability to oxidize three hexose alcohols and three disaccharides (Hayward, 1964), four phylotypes based on internal transcribed spacer (ITS) sequence analysis (Fegan and Prior, 2005), and at least 60 sequevars based on endoglucanase (*egl*) gene sequence similarities (Fegan and Prior, 2005). In 2014, phylotype II strains of the *R. solanacearum* species complex were classified as *R. solanacearum*, phylotypes I and III clustered into *R. pseudosolanacearum*, the phylotype I strains were named as a novel species *R. nicotianae* (Liu et al., 2023), and the phylotype IV strains were classified as *R. syzygii* in Safni et al. (2014). *R. syzygii* was initially proposed as *Pseudomonas syzygii* by Yabuuchi et al. (1995) before being transferred into the genus *Ralstonia* in 2004 and further sub-classified into three subspecies, *R. syzygii* subsp. *syzygii*, *R. syzygii* subsp. *indonesiensis* and *R. syzygii* subsp. *celebesensis* (Safni et al., 2014).

Bacteria in the genus *Ralstonia* are common pathogens in animals and plants (Paudel et al., 2020). The type strains of *R. pickettii*, *R. insidiosa*, and *R. mannitolilytica* are pathogenic to humans (Ralston et al., 1973; De Baere et al., 2001; Coenye et al., 2003). *R. pseudosolanacearum* and *R. solanacearum* are common pathogens causing soil-borne diseases in diverse crops, such as tobacco, tomato, and clove (Yabuuchi et al., 1995; De Baere et al., 2001; Coenye et al., 2003; Remenant et al., 2010, 2011; Safni et al., 2014; Zulperi et al., 2014; Lu et al., 2021). Bacteria belonging to the species *R. syzygii* are the causal agents of Sumatra disease of clove, blood disease of banana, and bacterial wilt of tobacco (Roberts et al., 1990; Ray et al., 2021; Lu et al., 2022a).

Although several species of the genus *Ralstonia* causing human and plant diseases have been studied widely, the non-pathogenic soil *Ralstonia* spp. have rarely been described. In the present study, 16S rRNA gene sequencing was performed on isolates from six tobacco-planting soils sampled in Yunnan, PR China, which identified eight *Ralstonia* strains. Combining genetic comparisons and phenotypic and chemotaxonomic characterizations, we provide evidence that these strains represent one known species *R. wenshanensis* and three novel *Ralstonia* species, proposed as *Ralstonia chuxiongensis* sp. nov. (type strain 21YRMH01-3^T^), *Ralstonia mojiangensis* sp. nov. (21MJYT02-10^T^), and *Ralstonia soli* sp. nov. (21MJYT02-11^T^).

## Materials and methods

### Sampling sites and bacterial strains

The eight strains were isolated from tobacco-planting soils collected from six sites in Yunnan, PR China: strain 21YRMH01-3^T^ was isolated from a tobacco field (101.550269 E, 26.071688 N; 1713 m above sea level) in Yongren, Chuxiong in May 2021; strains 21MJYT02-10^T^ and 21MJYT02-11^T^ were obtained from a tobacco field (101.47583 E, 23.18469 N; 1,630 m above sea level) in Mojiang, Puer in June 2021; strain 21LDWP02-16 was isolated from a tobacco field (103.548778 E, 27.137706 N; 2009.3 m above sea level) in Ludian, Zhaotong in May 2021; strain 22TCCZM01-4 was isolated from a tobacco field (98.661811 E, 24.825125 N; 1,192 m above sea level) in Tengchong, Baoshan in June 2022; strains 22TCJT01-1 and 22TCJT01-2 were isolated from a tobacco field (98.627645 E, 25.408019 N; 1,474 m above sea level) in Tengchong, Baoshan in June 2022; and strain 22TCCZM03-6 isolated from a tobacco field (98.682637 E, 24.858838 N; 1,222 m above sea level) in Tengchong, Baoshan in June 2022 (Supplementary Table S1). Previously described soil bacteria isolation and cultivation methods were employed (Lu et al., 2022a). The isolates were streaked onto trypticase soy agar (TSA, BD Difco, San Jose, CA, USA) at 28°C and purified three times by further plating. The isolates were then routinely cultured on TSA plates and stored for long-term preservation in 20% (v/v) glycerol at −80°C. For phenotypic and chemotaxonomic characteristic comparisons, four type strains, including *R. wenshanensis* 56D2^T^, *R. pickettii* JCM 5969^T^, *R. mannitolilytica* JCM 11284^T^, and *R. insidiosa* LMG 21421^T^, were used as references. Strains 21YRMH01-3^T^, 21MJYT02-10^T^, and 21MJYT02-11^T^ have been deposited at the Guangdong Microbial Culture Collection Center with the accession numbers GDMCC 1.3534^T^, GDMCC 1.3531^T^ and GDMCC 1.3532^T^, respectively, and at the Japan Collection of Microorganisms, with accession numbers JCM 35818^T^, JCM 35816^T^, and JCM 35817^T^, respectively.

### Phylogenetic and genomic analyses

Total DNA was extracted using a bacterial DNA isolation kit (catalog number D3107; GBCBIO Technologies Inc., Guangzhou, China). The 16S rRNA gene was amplified using the universal primer pair 27F/1492R (Clayton et al., 1995). The amplicons were ligated separately into vector pMD19-T (Takara, Dalian, China), sequenced following the Sanger method at Sangon Biotech Inc. (Shanghai, China), and assembled using SnapGene v6.0.2.^2^ The 16S rRNA gene sequences were compared with data in the EzBiocloud database (Yoon et al., 2017). The 16S rRNA gene sequences from each type species of the genera *Ralstonia* and *Cupriavidus* were collected from the GenBank database (Sayers et al., 2021), aligned using the MUSCLE program in Unipro UGENE v33.0 and trimmed to the same length for sequence identity calculation using the Clustal Omega tool in the EMBL-EBI database (Edgar, 2004; Sievers et al., 2011; Okonechnikov et al., 2012; Madeira et al., 2022). Phylogenetic trees were constructed using the neighbor-joining (NJ), maximum-likelihood (ML), and maximum-parsimony (MP) algorithms in the MEGA v11.0.13 software (Kumar et al., 2016). Bootstrap analysis was performed using 1,000 replications.

The EZNA Bacterial DNA Kit (Omega Bio-tek, Winooski, VT, USA) was employed for genomic DNA extraction. Genome sequencing, assembly, and annotation were conducted as detailed in our previous report (Lu et al., 2022b). We retrieved the assembly numbers for 649 genomic sequences of the genus *Ralstonia* from the GenBank database (collected on 2022-09-10) (Sayers et al., 2021). For quick detection, FastANI v1.33 (Jain et al., 2018) was used (kmer 16, fragment length of 3,000, and the minimal fraction was 0.2) to test the average nucleotide identity (ANI) values between the eight isolated strains and the above collected genomic sequences by employing the webserver genome taxonomy database (GTDB) (Parks et al., 2021). The ANIb (based on the BLAST algorithm), ANIm (based on the MUMmer algorithm), and digital DNA–DNA hybridization (dDDH) values between the eight isolated strains and their closely related species were calculated by using the webserver JSpecies WS v3.9.6 (Richter et al., 2015) and the Genome-to-Genome Distance Calculator v3.0 (Meier-Kolthoff et al., 2022), respectively. In addition, the Type (Strain) Genome Server (TYGS) was employed for phylogenomic analysis (Meier-Kolthoff et al., 2022).

To clarify the taxonomic position of the genus *Ralstonia*, all genome sequences of the validly published type species within the family *Burkholderiaceae* and those of closely related species were used for phylogenomic analysis. We collected a set of 288 genome sequences from the GenBank database, including 280, 6, and 2 genomes from the order *Burkholderiales*, *Neisseriales*, and *Rhodocyclales* of the class *Betaproteobacteria*, respectively. In addition, four genome sequences from the classes *Alphaproteobacteria* and *Gammaproteobacteria* were used as outgroups. Protein-coding sequences were predicted using the Prokka program (Seemann, 2014) and used to generate single-copy genes by employing proteinortho6 (Lechner et al., 2011). We used the diamond program for sequence alignment with the e-value cut-off set at 1e-5, a minimum aligned sequence length coverage of 50%, and an identity of 50% of the query sequence (Buchfink et al., 2021). A set of 92 single-copy gene clusters was concatenated using MUSCLE (Edgar, 2004). FastTree v2.0.0 with the default model Jones-Taylor-Thornton was used to construct the phylogenomic tree (Price et al., 2010), which was visualized and edited using FigTree v1.4.4.^3^

For gene content analysis, we identified the orthologous gene clusters in the genome sequences of seven non-plant pathogenic *Ralstonia* strains using the OrthoVenn2 web server (last accessed February 2023) with default settings (e-value 1e-5, inflation value 1.5) (Xu et al., 2019). The secondary metabolites and antibiotic gene clusters were analyzed *in silico* using the online web server antiSMASH bacterial v6.1.1, with detection strictness set as “relaxed” (Blin et al., 2021). Potential antibiotic resistance genes were identified using the Resistance Gene Identifier software against the Comprehensive Antibiotic Resistance Database using the protein homolog model (Alcock et al., 2022).

### Phenotypic and chemo-taxonomic characteristics

Several media, including casamino acid peptone glucose (CPG) agar (Kelman, 1954), Kelman’s tetrazolium chloride (TZC) (García et al., 2019), Luria-Bertani (LB) agar (LA), modified selective medium South Africa (mSMSA) agar (Elphinstone et al., 1996), nutrient agar (NA), potato dextrose agar (PDA), and TSA, were used for cell growth analysis at 28°C for 5 days. For strains’ antimicrobial susceptibility testing, antibiotics were supplemented in TSB at the following concentrations: ampicillin (100 μg/mL), bacitracin (10 μg/mL), chloromycetin (30 μg/mL), ciprofloxacin (1 μg/mL), cycloheximide (10 μg/mL), gentamicin (100 μg/mL), kanamycin (50 μg/mL), polymyxopeptide (10 μg/mL), rifamycin (25 μg/mL), streptomycin (100 μg/mL), and tetracycline (15 μg/mL). Cells grown on TSA at 28°C for 48 h were subjected to morphological observation using transmission electron microscopy (HT7700; HITACHI, Tokyo, Japan). The motilities of the tested strains were detected on modified semi-solid motility media (SMM) containing 0.35% agar (Kelman and Hruschka, 1973). A staining kit (D008-1-1; Nanjing Jiancheng Bioengineering Institute, Nanjing, China) was used for Gram-staining, which was detected under a light microscope (Axio imager.Z2; Zeiss, Oberkochen, Germany). For the oxygen requirement assay, strains were streaked on TSA slopes covered with mineral oil and incubated at 28°C for 5 days (Lu et al., 2022b).

Strains cultured on TSA were collected to compare their physiological and biochemical features. Substrate utilization and enzymatic activities were tested according to the manufacturer’s instructions for the API 20NE and API ZYM kits (bioMérieux, Marcy-l’Étoile, France). Oxidase activities of strains were tested using 1% (w/v) tetramethyl-*p*-phenylenediamine, with a color change to dark blue within 90 s regarded as positive. The production of bubbles detected catalase activity after adding a drop of 3% (v/v) H_2_O_2_ (Lu et al., 2022a). According to the manufacturer’s instructions, carbon source utilization and chemical sensitivity were determined using the Biolog GEN III Microplate system (Biolog, Hayward, CA, USA). Cells of all tested strains that grew on TSA at 28°C for 48 h were harvested for fatty acid profile analysis, as described by Sasser (1990). Polar lipids of three proposed type strains were extracted and identified using a modified version of a previously published method (Minnikin and Abdolrahimzadeh, 1971). In brief, the cells were grown at 28°C in 2 L of nutrient broth (NB) for 48 h, collected by centrifugation at 6000 × *g* for 10 min, and washed 2–3 times with distilled water. Then, 1 g of wet bacteria was placed in a 40 mL centrifuge tube with a screw cap, added with 15 mL of methanol to suspend the pellet, and then heated in a water bath at 100°C for 10 min. After cooling to room temperature, 10 mL of chloroform was added into the tube, followed by the slow addition 2% (w/v) NaCl until stratification. The tube was shaken vigorously for 10 min and centrifuged at 8000 × *g* for 10 min to complete the phospholipid precipitation. The tube was left to stand vertically, and after phase separation, the liquid layer without impurities was removed into a round-bottom rotary evaporator flask and placed in a water bath at 30–40°C to accelerate drying. After drying, 400 μL of chloroform/methanol (2:1, v/v) was added to dissolve the lipids. Then the liquid was added into a microfuge tube (approximately 100–200 μL/tube) and centrifuged at 8000 × *g* for 10 min to remove the pellet. If the solution was layered, the layer with bubble was removed, and 5–15 μL of the sample was spotted at 1.5 × 1.5 cm from the bottom left-hand corner of a Silica gel 60 plate (Merck, Darmstadt, Germany). The plates were developed in two dimensions: The first dimension contained chloroform:methanol:distilled water (65,25:4, v/v/v), and the second dimension contained chloroform:acetic acid:methanol:distilled water (80,18,12:5,v/v/v/v). The first dimension was run from the spotting site to 1.5 cm away from the plate’s leading edge, after which the plate was allowed to dry at room temperature for about 30 min. The second dimension was run to 1.5 cm away from the top edge of the plate and then allowed to dry at room temperature for about another 30 min. Reagents, including ninhydrin, molybdenum blue, α-methyl naphthol and D reagent, were sprayed on the plates to identify lipid functional groups. The plates were sprayed with 10% (v/v) molybdophosphoric acid to show all polar lipids. The isoprenoid quinones of the three proposed type strains were extracted and analyzed as described previously (Collins and Jones, 1981).

## Results and discussion

### 16S rRNA gene phylogeny

**Figure 1:**
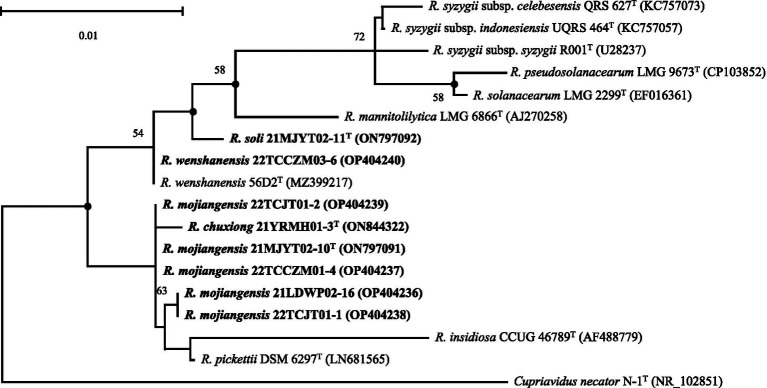
Maximum-likelihood phylogenetic tree based on 16S rRNA sequences (1,397 bp) showing the relationship between soil strains isolated in this study and their closely related taxa within the genus Ralstonia. The tree was generated using MEGA 11.0. Bootstrap values (≥50) are shown at branching points as percentages of 1,000 replicates. ^T^ indicates the type strain. Closed circles indicate that the corresponding nodes are also recovered in the trees generated with neighbor-joining and maximum-parsimony. Sequences from soil strains isolated in this study are indicated in bold. *Cupriavidus necator* N-1^T^ (Poehlein et al., 2011) is used as an outgroup. The NCBI accession numbers are shown in parentheses. Bar, 0.01 substitutions per nucleotide position.

During a cultivation-based analysis of bacteria from tobacco-planting soils collected from four cities in Yunnan, PR China, eight closely related *Ralstonia* spp. strains were isolated (Supplementary Table S1). The 16S rRNA gene sequences of the eight strains (21YRMH01-3^T^, 21MJYT02-10^T^, 21LDWP02-16, 22TCCZM01-4, 22TCJT01-1, 22TCJT01-2, 21MJYT02-11^T^, and 22TCCZM03-6) were obtained for pair-wise comparisons. The analysis showed 98.34–100.0% similarity between each pair among the isolates. The 16S rRNA gene sequences were identical for strains 21MJYT02-10^T^, 22TCCZM01-4, and 22TCJT01-2, and were 99.30% similar to that of strain 21LDWP02-16, which shared an identical 16S rRNA gene with strain 22TCJT01-1, suggesting that these five strains might represent the same species. A sequence similarity search in the Ezbiocloud database showed that the eight isolated strains were closely related to the type species of the genus *Ralstonia*. Strain 22TCCZM03-6 shared an identical 16S rRNA gene sequence with *R. wenshanensis* 56D2^T^, indicating that these strains might be the same species. The other seven strains had the highest 16S rRNA gene sequence identities to those of *R. wenshanensis* 56D2^T^ (98.70–99.64%), *R. pickettii* K-288^T^ (Daligault et al., 2014) (98.34–99.86%), *R. insidiosa* LMG 21421^T^ (97.34–98.56%), and *R. mannitolilytica* LMG 6866^T^ (98.35–98.49%). The 16S rRNA gene sequence similarities between the eight isolated strains and other type species of the genus *Ralstonia* were below the species cut-off value of 98.50% (Chun et al., 2018; Supplementary Table S2). The ML phylogenetic tree showed that the eight isolated strains fell within the clade comprising species of the genus *Ralstonia* and were closely related to *R. wenshanensis* 56D2^T^, *R. pickettii* K-288^T^, *R. insidiosa* LMG 21421^T^, and *R. mannitolilytica* LMG 6866^T^ (Figure 1 and Supplementary Figures S1, S2).

### Genome features

The draft genome lengths of the eight isolated strains ranged from 5.17 to 5.72 Mb and contained 4,712–5,191 annotated protein-coding genes. The overall G + C contents of the eight genomes were 63.3–64.1%, which were close to three related species type strains of *R. wenshanensis* 56D2^T^ (63.7%), *R. insidiosa* LMG 21421^T^ (63.7%), and *R. pickettii* K-288^T^ (63.9%), but markedly lower than those of *R. mannitolilytica* LMG 6866^T^ (65.8%) and plant-pathogenic *Ralstonia* species including *R. syzygii* subsp. *indonesiensis* LMG 27703^T^ (66.3%), *R. syzygii* LLRS-1 (66.3%), *R. solonacearum* K60-1^T^ (Remenant et al., 2012) (66.4%), *R. syzygii* subsp. *syzygii* LMG 10661^T^ (66.5%), *R. syzygii* subsp. *celebesensis* UGMSS_Db01 (Prakoso et al., 2022) (66.6%), and *R. pseudosolanacearum* LMG 9673^T^ (66.6%) (Supplementary Table S3).

Genomic comparisons based on ANI and dDDH values were calculated between the eight isolated strains and their close relatives within the genus *Ralstonia* (Table 1). When compared with strain 22TCCZM03-6, *R. wenshanensis* 56D2^T^ shared ANIb, ANIm, and dDDH values of 98.08, 98.29, and 84.80%, respectively, showing that these strains are the same species, which was consistent with the results from the 16S rRNA gene analysis. When compared with strain 21YRMH01-3^T^, *Burkholderiaceae* bacterium 26, isolated from soil in 2010 in the USA (Roco et al., 2017), had ANIb, ANIm, and dDDH values of 97.22, 97.65, and 78.90%, respectively. Both ANI and dDDH values estimated for other tested strains were less than 94.5 and 55.5%, which are below the proposed species cut-off values of 95–96% for ANI and 70% for dDDH, indicating that strains 21YRMH01-3^T^ and *Burkholderiaceae* bacterium 26 represent a novel species in the genus *Ralstonia*. When strain 21MJYT02-11^T^ was compared, the ANI and dDDH values were 73.75–88.33% and 22.10–33.20%, respectively, supporting the inclusion of this strain as a novel species separated from validated and published *Ralstonia* species. When strain 21MJYT02-10^T^ was compared, four isolates, including 21LDWP02-16, 22TCJT01-1, 22TCCZM01-4, and 22TCJT01-2, had ANI values >95% and dDDH values >70% (yielded by formula 2). The ANI and dDDH values calculated for the other analyzed strains were 73.32–94.17 and 22.0–55.20, respectively, supporting the classification of 21MJYT02-10^T^, 21LDWP02-16, 22TCJT01-1, 22TCCZM01-4, and 22TCJT01-2 as a novel species in the genus *Ralstonia*.

**Table 1:** Genomic comparisons of *Ralstonia* spp. strains isolated in this study and their closely related type strains.

Strain	21YRMH01-3^T^			21MJYT02-10^T^			21MJYT02-11^T^			*R. wenshanensis* 56D2^T^		
	ANIb	ANIm	dDDH	ANIb	ANIm	dDDH	ANIb	ANIm	dDDH	ANIb	ANIm	dDDH
*Ralstonia chuxiongensis* 21YRMH01-3^T^	100.00	100.00	100.00	93.41	94.17	55.20	86.23	88.23	33.00	93.05	93.73	53.50
*R. chuxiongensis* 26	97.22	97.65	78.90	93.42	94.19	55.50	93.07	93.75	32.90	86.28	88.18	53.40
*R. mojiangensis* 21MJYT02-10^T^	93.57	94.17	55.20	100.00	100.00	100.00	86.38	88.22	33.10	92.30	93.08	50.10
*R. mojiangensis* 21LDWP02-16	93.54	94.20	55.40	98.45	98.70	88.50	86.29	88.23	33.10	92.33	93.06	50.20
*R. mojiangensis* 22TCJT01-1	93.54	94.20	55.30	98.45	98.69	88.10	86.40	88.25	33.20	92.32	93.07	50.30
*R. mojiangensis* 22TCCZM01-4	93.55	94.15	55.00	98.11	98.55	86.30	86.34	88.17	33.00	92.47	93.12	50.40
*R. mojiangensis* 22TCJT01-2	93.41	94.13	55.00	98.06	98.49	85.90	86.34	88.21	33.00	92.36	93.11	50.50
*R. wenshanensis* 56D2^T^	93.35	93.73	53.50	92.47	93.08	50.10	86.54	88.26	33.10	100.00	100.00	100.00
*R. wenshanensis* 22TCCZM03-6	93.44	93.74	53.40	92.60	93.07	50.10	86.55	88.21	33.00	98.08	98.29	84.80
*R. pickettii* K-288^T^	90.74	91.56	44.00	91.01	91.74	44.40	86.36	88.11	32.70	90.70	91.66	44.30
*R. soli* 21MJYT02-11^T^	86.03	88.23	33.00	85.92	88.22	33.10	100.00	100.00	100.00	86.02	88.26	33.10
*R. mannitolilytica* LMG 6866^T^	85.84	87.68	31.00	85.99	87.77	31.20	85.89	87.92	31.70	86.02	87.89	31.60
*R. insidiosa* LMG 21421^T^	84.89	87.51	30.60	84.79	87.47	30.40	85.62	88.33	32.30	84.83	87.54	30.70
*R. pseudosolanaecearum* LMG 9673^T^	82.37	86.22	26.40	82.36	86.28	26.30	83.20	86.87	27.70	82.56	86.38	26.70
*R. syzygii* subsp. *syzygii* LMG 10661^T^	82.79	86.16	26.40	82.81	86.13	26.50	83.61	86.65	27.60	82.90	86.21	26.70
*R. solanacearum* K60-1^T^	82.19	86.10	26.20	82.15	86.09	26.10	83.08	86.66	27.20	82.54	86.17	26.50
*R. syzygii* subsp. *indonesiensis* LMG 27703^T^	82.09	86.12	26.20	82.13	86.15	26.20	83.02	86.68	27.50	82.31	86.20	26.60
*Cupriavidus necator* N-1^T^	73.38	84.63	21.70	73.32	84.66	22.00	73.75	84.71	22.10	73.53	84.77	21.90

The phylogenomic analysis revealed that the genomic tree was divided into two branches, one containing the eight isolated strains and their closely related species of *R. wenshanensis*, *R. pickettii*, *R. mannitolilytica*, and *R. insidiosa*; and the other one harboring three species of *R. syzygii*, *R. pseudosolanacearum*, and *R. solanacearum*. In branch one, strains 22TCCZM03-6 and *R. wenshanensis* 56D2^T^ were positioned separately from the other species of the genus *Ralstonia* and formed a clade with a support value of 100%; five strains (21MJYT02-10^T^, 22TCJT01-1, 21LDWP02-16, 22TCJT01-2, and 22TCCZM01-4) formed a coherent cluster with strains 21YRMH01-3^T^ and *Burkholderiaceae* bacterium 26, with a bootstrap value of 82%. In addition, strain 21MJYT02-11^T^ was placed independently of the other *Ralstonia* species and formed a monophyletic clade with a bootstrap value of 75% (Supplementary Figure S3).

**Figure 2:**
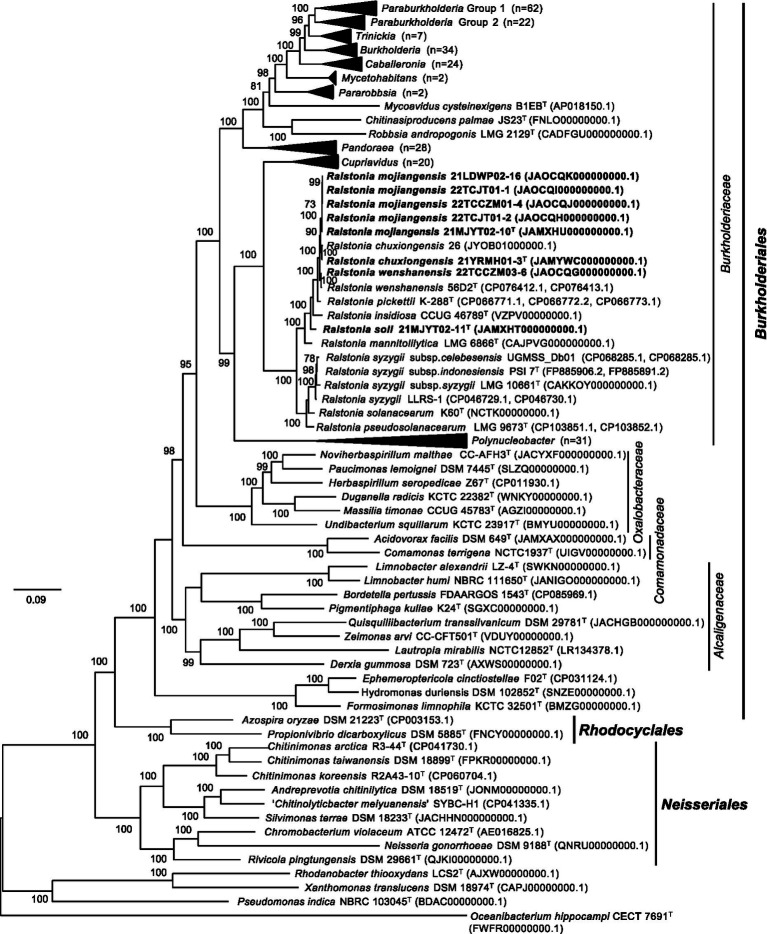
The phylogenomic tree based on the concatenation of 92 single-copy genes clusters showing the relationship between the genus *Ralstonia* and other genera within the order *Burkholderiales*. Protein-coding sequences were predicted by the Prokka program (Seemann, 2014) and used to generate single-copy genes by employing proteinortho6 (Lechner et al., 2011). The diamond program was run for sequence alignment with a set *e*-value cut-off of 1e-5, a minimum aligned sequence length coverage of 50%, and an identity of 50% of the query sequence (Buchfink et al., 2021). A set of 92 single-copy gene clusters was concatenated with MUSCLE v5.1 (Edgar, 2004). FastTree v2.0.0 with the default model Jones-Taylor-Thorton (Price et al., 2010) was used to construct the phylogenomic tree, which was visualized and edited by the FigTree v1.4.4 (http://tree.bio.ed.ac.uk/software/figtree/). The tree is rooted at the midpoint. Bootstrap values (>50) are shown at branch nodes as percentages of 1,000 replications. ^T^ indicates the type strain. Sequences from soil strains obtained in this study are indicated in bold. The *Rhodanobacter thiooxydans* LCS2^T^, *Xanthomonas translucens* DSM 18974^T^ and *Pseudomonas indica* NBRS 103045^T^ of the class *Gammaproteobacteria*, and *Oceanibacterium hippocampi* CECT 7691^T^ of the class *Alphaproteobacteria* are used as outgroups. Genomic accession numbers are shown in parentheses. The scale bar indicates the number of substitutions per site.

To clarify the taxonomic position of the genus *Ralstonia*, a total of 92 conserved single-copy genes were identified among 288 tested genomes. Phylogenomic analysis showed that all strains isolated in this study and the type species in the genus *Ralstonia* formed a single branch containing two subbranches, consistent with the results obtained from the above phylogenomic analysis. Interestingly, the bacteria from the genera *Cupriavidus* and *Polynuclearbacter*, along with those from the genus *Ralstonia* formed a coherent super branch with other genera of the family *Burkholderiaceae* (Figure 2), suggesting that strains within the former group might present a novel family.

In addition, evolutionary analyses revealed that several genera belonging to the family *Burkholderiaceae* were clustered with the members of the other families in the class *Betaproteobacteria*. The type species *Paucimonas lemoignei* DSM 7445^T^ was clustered with *Noviherbaspirillum malthae* CC-AFH3^T^ of the family *Oxalobacteraceae*, which was consistent with the results obtained from a previous report (Ludwig et al., 2021). Five type strains of the family *Burkholderiaceae*, including *Limnobacter alexandrii* LZ-4^T^, *Limnobacter humi* NBRC 111650^T^, *Quisquiliibacterium transsilvanicum* DSM 29781^T^, *Zeimonas arvi* CC-CFT501^T^, and *Lautropia mirabilis* NCTC12852^T^, were posited in the family *Alcaligenaceae*. All three genome-sequenced type strains, including *Chitinimonas taiwanensis* DSM 18899^T^, *Chitinimonas koreensis* DSM17726^T^, and *Chitinimonas arctica* R3-44^T^, were clustered with members of the family *Chromobacteriaceae* in the order *Neisseriales*, which was consistent with a previous report (Chen et al., 2021), in which the type species *Chitinimonas taiwanensis* was assigned to the family *Chitinibacteraceae*. Furthermore, three recently proposed type strains, including *Ephemeroptericola cinctiostellae* F02^T^ (Kim et al., 2019), *Hydromonas duriensis* DSM 102852^T^ (Vaz-Moreira et al., 2015), *Formosimonas limnophila* KCTC 32501^T^ (Chen et al., 2017), formed an independent branch in the order *Burkholderiales* (Figure 2). The above data suggested that these species might need to be reclassified to other families or orders.

### Pan-genome and accessory genes analysis

The genome sequence predictions showed that seven non-plant pathogenic *Ralstonia* strains had a set of 35,382 protein-coding genes (average 5,055 genes/species). Pairwise genome comparisons using the OrthoVenn2 web server identified 5,828 gene clusters, including 2,761 orthologous clusters and 3,067 single-copy gene clusters. Further analysis showed that 3,112 orthologous genes were shared by all tested strains, indicating their conservation in the lineage after speciation. These core orthologous genes potentially participate in critical metabolic processes for nitrogen compounds, cellular aromatic compounds, nucleobase-containing compounds, organic acids, heterocyclic compounds, macromolecules, phosphorus, and cellular lipids. In addition, the data showed that strain 21YRMH01-3^T^ shared 123 specific gene clusters with 21MJYT02-10^T^, 171 distinct clusters from strain 21MJYT02-11^T^ were shared with *R. insidiosa* CCUG 46789^T^, and 87 unique gene clusters from *R. wenshanensis* 56D2^T^ were shared with 21YRMH01-3^T^, suggesting that these strains are closely related to each other.

**Figure 3:**
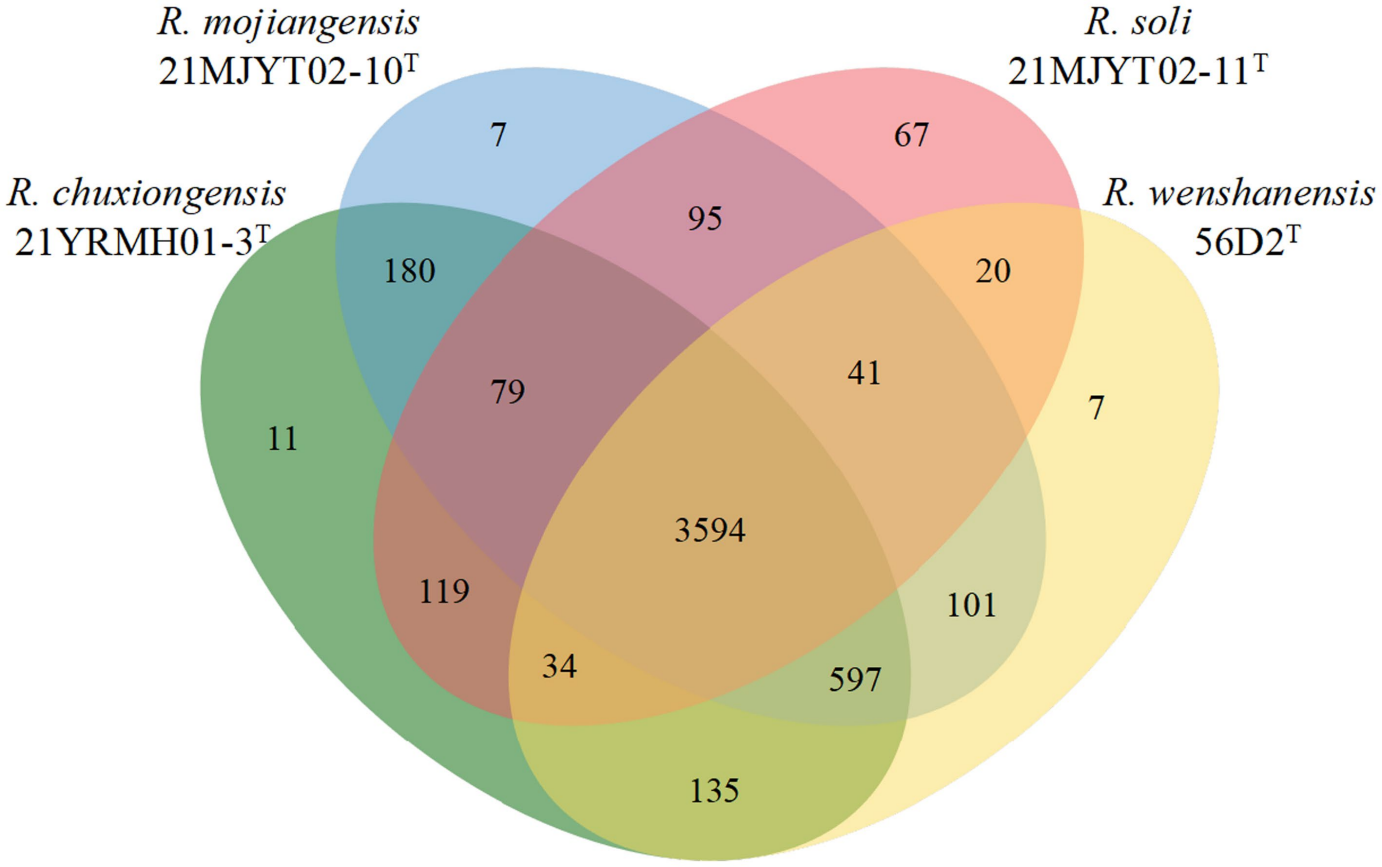
Venn diagram displays the distribution of shared orthologous clusters among the four soil *Ralstonia* strains. Protein-coding sequences were predicted by the Prokka program (Seemann, 2014). The orthologous gene clusters were identified using the OrthoVenn2 web server, with a threshold e-value of 1e-5 and inflation of 1.5 (Xu et al., 2019).

Furthermore, protein-coding genes of four *Ralstonia* strains isolated from tobacco planting soils, including 21YRMH01-3^T^, 21MJYT02-10^T^, 21MJYT02-11^T^, and *R. wenshanensis* 56D2^T^, were compared. The diagram showed that these four strains shared 3,594 core orthologous gene clusters, and there were 11, 7, 67, and 7 gene clusters specific to strains 21YRMH01-3^T^, 21MJYT02-10^T^, 21MJYT02-11^T^, and *R. wenshanensis* 56D2^T^, respectively. In strain 21YRMH01-3^T^, one out of 11 gene clusters encoded two proteins that shared sequence similarity to the iron–sulfur subunit SdhB of succinate dehydrogenase, which is used in aerobic growth in *Escherichia coli* K-12 (Darlison and Guest, 1984); however, the functions for other 10 gene clusters (encoding 20 proteins) remain unknown. In strain 21MJYT02-10^T^, there were three and two proteins that might be involved in the regulation of DNA-templated transcription (GO:0006355) and ethanol oxidation (GO:0006069), respectively; the other five gene clusters (encoding 11 proteins) shared no sequence similarity with the proteins deposited in the Swiss-Prot database (Figure 3). In strain 21MJYT02-11^T^, there were 67 unique gene clusters encoding 150 proteins, which are mainly involved in the biological process, metabolic process, cellular metabolic process, and cellular process; however, the functions of 50 of these proteins are unknown.

### *In silico* prediction of secondary metabolites

Genome sequence scanning showed that gene clusters encoding secondary metabolites, such as terpene, unspecified ribosomally synthesized and post-translationally modified peptide product (RiPP-like), aryl polyene, siderophore, beta-lactone containing protease inhibitor (betalactone), and redox-cofactor, were present in most analyzed strains (Supplementary Table S4). The first five putative secondary metabolite-encoding clusters were conserved in two completely sequenced strains, *R. wenshanensis* 56D2^T^ and *R. pickettii* K-288^T^. These gene clusters might also be widespread among other non-plant pathogenic *Ralstonia* strains. Only RiPP-like gene clusters were identified from the megaplasmid of the completely sequenced strains, suggesting that other gene clusters were obtained through vertical transfer. Certain strains even contained two homologs of putative metabolite synthetic gene clusters, such as *R. mannitolilytica* LMG 6866^T^, 21MJYT02-10^T^, and 21MJYT02-11^T^, which had two copies of the aryl polyene cluster in each strain; and strains 21YRMH01-3^T^ and 21MJYT02-10^T^, which had two copies of the betalactone and siderophore clusters, respectively. The putative redox-cofactor gene clusters, with a sequence similarity of 13% to that of lankacidin C, were identified in all analyzed strains, except *R. pickettii* K-288^T^. However, a type I polyketide synthase gene cluster, shared 8% sequence similarity with the lipopolysaccharide cluster, was identified in *R. pickettii* strain K-288^T^.

### Resistome and antibiotic susceptibility

Antibiotic-resistance gene analysis identified 46 antibiotic-resistance genes in 10 tested strains. More resistance genes were found in the genomes of non-plant pathogenic *Ralstonia* (average 5.14 genes per strain) than in plant pathogenic strains (average 3.33 genes per strain) (Supplementary Table S5). Antimicrobial susceptibility analysis confirmed that most non-phytopathogenic bacteria of the *Ralstonia* were resistant to all tested antibiotics except ciprofloxacin (Supplementary Table S6). By contrast, plant pathogenic *Ralstonia* species were only resistant to four tested antibiotics, such as bacitracin, chloromycetin, cycloheximide, and polymyxopeptide. The adeF efflux pump genes, which contribute to fluoroquinolone and/or tetracycline resistance (Coyne et al., 2010), were conserved among the type strains. Non-plant pathogenic strains had two copies of a protein, one shared about 79% sequence similarity, and another one had 43% sequence similarity to AdeF protein (antibiotic resistance gene ontology (ARO) No. 3000777), except for *R. wenshanensis* 56D2^T^, which harbored three copies of this gene. However, the genomes of all tested plant pathogenic *Ralstonia* strains had three copies of AdeF. Interestingly, all tested strains were susceptible to ciprofloxacin in antibiotic-sensitive assay. Furthermore, the *tetD* gene, encoding a tetracycline resistance protein (ARO No. 3000168, about 43% protein sequence identity), was found in strains including 21YRMH01-3^T^, 21MJYT02-10^T^, *R. insidiosa* CCUG 46789^T^, and *R. wenshanensis* 56D2^T^, and the resistance phenotype of these strains to tetracycline was confirmed in antimicrobial susceptibility assay.

Notably, one or two copies of genes, whose encoded proteins showed more than 86% sequence similarity, encoded OXA (oxacillin-hydrolyzing) beta-lactamases (ARO No. 3001417, 3003599, 3003600, 3005793, 3005326, 3005325, 3005795, 3001808, and 3005093) confer resistance to amino- and ureidopenicillin (Evans and Amyes, 2014) and were present in all non-plant pathogenic strains. The *R. soli* 21MJYT02-11^T^ genome revealed the presence of a unique glycopeptide resistance protein VanH (ARO: 3002942, 38.54% protein sequence identity). In addition, the small multidrug resistance (SMR) antibiotic *qacG/J* efflux pump genes (ARO No. 3007014 and 3007015, about 44% protein sequence identity) were identified from *R. syzygii* subsp. *indonesiensis* PSI 7^T^, *R. insidiosa* CCUG 46789^T^, and *R. mannitolilytica* LMG 6866^T^ (Supplementary Table S5).

Antimicrobial susceptibility testing showed that all tested strains of the genus *Ralsotnia* were resistant to polymyxopeptide, cycloheximide, bacitracin, and chloromycetin. However, most phytopathogenic strains of *Ralstonia* were susceptible to kanamycin, tetracycline, streptomycin, rifamycin, ampicillin, and gentamicin. In addition, 50% of tested non-plant pathogenic strains were sensitive to tetracycline. Interestingly, strain 21MJYT02-11^T^ was susceptible to kanamycin, tetracycline, streptomycin, and gentamicin; however, other non-plant pathogenic strains of the *Ralstonia* were resistant or partially tolerant to these antibiotics (Supplementary Table S6).

### Morphology and physiology

**Figure 4:**
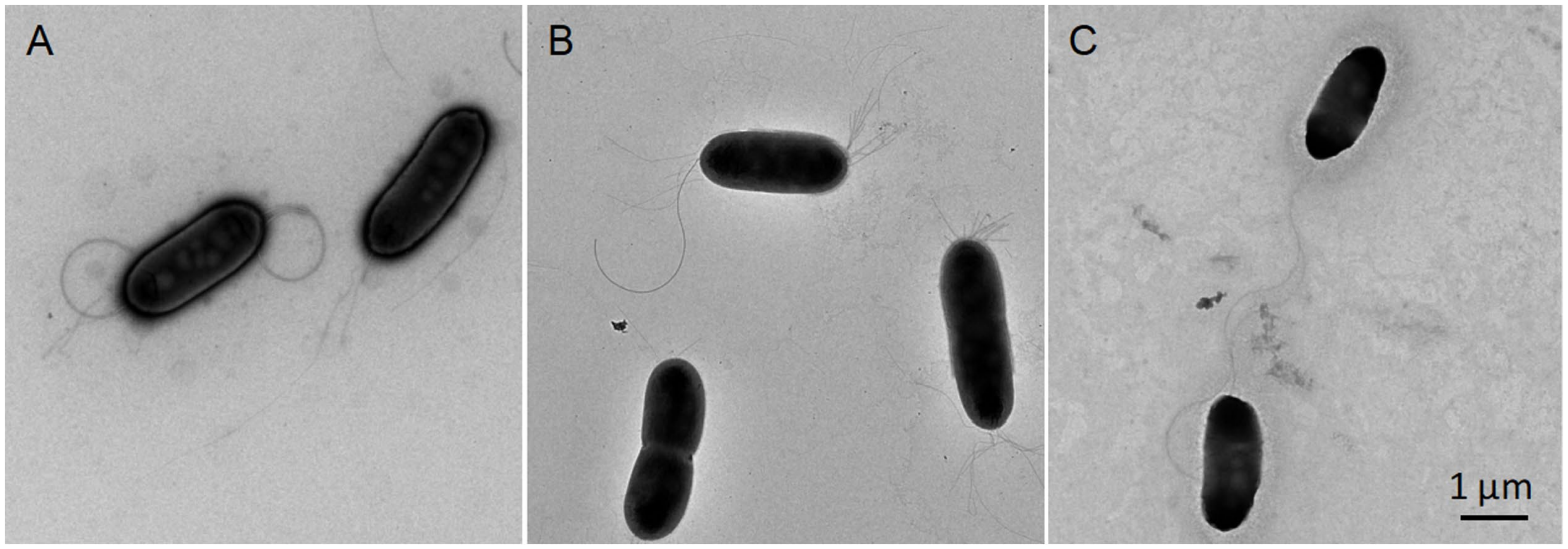
Transmission electron microscopy images showing the morphologies of three novel type strains of the genus *Ralstonia*. Cells grown on TSA media at 28°C for 48 h were subjected to morphological observation using transmission electron microscopy (HT7700; HITACHI, Tokyo, Japan). *R. chuxiongensis* 21YRMH01-3^T^ **(A)**; *R. mojiangensis* 21MJYT02-10^T^ **(B)**; *R. soli* 21MJYT02-11^T^ **(C)**. The bar represents 1 μm.

All strains could grow on CPG, LB, mSMSA, NA, PDA, TSA, and TZC, with better growth on CPG and TZC. Colonies were round, opaque, and raised, with glistening surfaces with entire edges and were 1.0–1.5 mm in diameter on TSA plates after 48 h of incubation at 28°C. Strains grew on 0–5% NaCl, but not on 6% (optimum 0%), and at 10–40°C, but not at 45°C (optimum 25°C). All strains were aerobic and motile on SMM, positive for catalase activities, and negative for oxidase tests. The cells were Gram-stain negative, aerobic, non-sporulating, motile with two polar flagella, and rod-shaped (Figure 4).

In the API 20NE and API ZYM tests, all tested strains were positive for the hydrolysis of esculin; for the enzymatic activities of esterase (C4), esterase lipase (C8), and leucine arylamidase, for the assimilation of D-glucose, potassium gluconate, and trisodium citrate; and negative for indole production; for the enzymatic activities of arginine dihydrolase, urease, trypsin, α-chymotrypsin, α-galactosidase, β-galactosidase, β-glucuronidase, α-glucosidase, β-glucosidase, N-acetyl-β-glucosaminidase, α-mannosidase, and α-fucosidase, and for the assimilation of D-mannose and D-maltose. For many of the phenotypic characteristics, the eight isolated strains were more similar to each other, such as being negative for fermentation of glucose, hydrolysis of gelatin, and enzymatic activity of cystine arylamidase; and positive for the assimilation of malic acid. However, strains 21YRMH01-3^T^ and 21MJYT02-11^T^ had several distinct phenotypic characteristics, such as being positive for alkaline phosphatase and acid phosphatase activities, and negative for the reduction of nitrate to nitrite. By contrast, most other strains had the opposite phenotypic characteristics. Strain 21YRMH01-3^T^ also had several unique phenotypic features that differed from strain 21MJYT02-11^T^. For example, strain 21YRMH01-3^T^ could not assimilate adipic acid and phenylacetic acid, but could assimilate L-arabinose and D-mannitol, and was positive for lipase activity (C14) (Table 2).

**Table 2:** Phenotypic traits differentiating strains obtained in this study and their closely related type strains of the genus *Ralstonia*.

Characteristics	1	2	3	4	5	6	7	8	9^*^	10^*^	11^*^	12^*^
Reduction of nitrate to nitrite (API 20NE)	−	−	+	+	+	+	−	+	+	+	−	−
Fermentation of glucose (API 20NE)	−	−	−	−	−	−	−	−	+	+	+	+
Hydrolysis of gelatin (API 20NE)	−	−	−	−	−	−	−	−	+	−	+	−
Assimilation of (API 20NE)												
Capric acid	+	+	−	+	+	+	+	+	+	−	+	+
Adipic acid	−	−	−	−	−	−	+	−	−	−	−	+
Malic acid	+	+	+	+	+	+	+	+	+	+	−	+
Phenylacetic acid	−	−	−	−	−	−	+	−	+	−	−	+
Enzyme activity of (API ZYM)												
Alkaline phosphatase	+	−	−	−	−	−	+	−	−	−	+	−
Lipase (C14)	+	+	+	+	+	+	−	+	+	−	+	+
Valine arylamidase	−	+	−	−	−	−	−	−	−	−	+	−
Cystine arylamidase	−	−	−	−	−	−	−	−	−	−	+	−
Acid phosphatase	+	−	−	−	−	−	+	−	−	−	−	−
Naphthol-AS-BI-phosphohydrolase	+	+	−	−	+	−	+	+	−	+	+	+
Utilization of (Biolog GEN III)												
Dextrin	−/+	−	−/+	−/+	−/+	−/+	−/+	−/+	−/+	−/+	−	−/+
D-trehalose	−/+	−	−	−/+	−/+	−/+	−	+	+	−	−	−
D-cellobiose	−	−	−	−	−	−/+	−	−/+	−	−	−	−
Gentiobiose	−	−	−	−	−	−/+	−	−/+	−/+	−	−	−/+
D-turanose	−	−	−	−	−	−	−	−	−	−/+	−/+	−
pH 6	+	+	−/+	+	+	+	+	+	+	−/+	+	−
pH 5	+	−/+	−	+	+	+	−/+	+	−	−	−	−
D-melibiose	−/+	−	−/+	−	−/+	−	−	−/+	+	−	−	−/+
D-salicin	−	−	−/+	−	−	−	−	−	−	−	−	−
N-acetyl-D-glucosamine	−/+	−/+	−/+	−/+	−/+	−/+	−/+	+	−	−	−	−
N-acetyl-β-D-mannosamine	−	−	−/+	−	−	−	−	−/+	+	−	−	−
D-mannose	−	−	−	−	−	−	−	−/+	−/+	−	−/+	−/+
3-methyl glucose	−/+	−	−/+	−/+	−/+	−/+	−	−/+	−/+	−	−/+	−/+
D-fucose	+	+	−/+	+	+	+	−/+	+	+	−	−/+	−/+
L-fucose	+	+	−/+	+	+	+	−/+	+	+	−	−	−/+
L-rhamnose	−/+	−	−	−/+	−/+	−/+	−	−/+	−	−	−	−
1% sodium lactate	+	+	+	+	+	+	+	+	+	−	−/+	+
Fusidic acid	+	−	−	+	−	+	−/+	+	−	−	−	−/+
D-serine	−	−/+	−/+	−	−	−	+	−/+	−/+	−	−	−
D-mannitol	−/+	−	−/+	−/+	−/+	−/+	−	−/+	−/+	−	−/+	−
D-arabitol	−/+	−	−/+	−/+	−/+	−/+	−	−/+	−/+	−	−	−
Glycerol	−/+	−	−/+	−	−/+	−/+	−/+	−/+	−	−	−/+	−/+
D-glucose- 6-PO_4_	−/+	−	−/+	−/+	−/+	−	−	−/+	−	−/+	−	−/+
D-aspartic acid	−	+	−	+	+	+	+	+	−/+	−	−	−/+
Troleandomycin	+	−	+	+	+	+	−	−	+	−	+	−/+
L-alanine	+	+	−	+	+	+	−/+	+	−/+	−	+	+
L-arginine	−	−	−	−	−	−	+	−	−	−	−	−
L-pyroglutamic acid	+	−	−/+	+	+	+	+	+	−/+	−/+	+	+
L-serine	+	−/+	−	+	+	+	−	+	−/+	−	−/+	−/+
Guanidine HCl	+	+	−	+	+	+	+	+	−/+	+	−/+	+
Niaproof 4	+	+	+	+	+	+	+	+	−/+	−	−	+
Pectin	−	−	−	−	−	−	−	−	−/+	−/+	−/+	−/+
L-galactonic acid lactone	+	+	+	+	+	+	−	+	+	−/+	+	−/+
D-gluconic acid	−/+	−	−	−/+	−/+	−/+	+	−/+	−/+	−/+	+	+
Mucic acid	+	−	−	−/+	−/+	+	−	+	−/+	−	+	−
Quinic acid	+	−/+	−	+	+	+	+	+	+	−	−	+
*p*-hydroxy-phenylacetic acid	−	−	−	−	−	−/+	−/+	−/+	−	−	−/+	−/+
Methyl pyruvate	−/+	−	−	−/+	−/+	+	−/+	−	−/+	−	−/+	+
D-lactic acid methyl ester	−	−	−	−/+	−	−	−	−	−	−	−	+
L-lactic acid	+	−/+	−/+	+	+	+	+	+	+	−	+	+
Citric acid	−/+	−	+	−/+	−/+	+	+	+	−	−/+	+	+
α-keto-glutaric acid	+	+	+	+	+	+	+	+	+	+	−	+
L-malic acid	−/+	−/+	+	+	+	+	+	+	−/+	−/+	−	+
Bromo-succinic acid	−	−	−/+	−	−	−	−	−	−	−	−	−/+
Nalidixic acid	+	+	+	+	+	+	+	+	−/+	−	+	−/+
Tween 40	+	+	+	+	+	+	+	+	+	−	+	+
γ-amino-butryric acid	−/+	−	−	−/+	−/+	−/+	+	−/+	−/+	−	+	+
α-hydroxy-butyric acid	−/+	−	−	−	−	−	−	−/+	−	−	−	−/+
α-keto-butyric acid	−	−	−	−	−	−/+	−	−	−	−	−	−
Propionic acid	+	−	+	+	+	+	−	+	−/+	+	−/+	+
Formic acid	−/+	−	−	−/+	−	−/+	−	−/+	−	−/+	−	−/+

Strains: 1. 21YRMH01-3^T^; 2. 21MJYT02-10^T^; 3. 21LDWP02-16; 4. 22TCCZM01-4; 5. 22TCJT01-1; 6. 22TCJT01-2; 7. 21MJYT02-11^T^; 8. 22TCCZM03-6; 9. *R. wenshanensis* 56D2^T^; 10. *R. pickettii* JCM 5969^T^; 11. *R. mannitolilytica* JCM 11284^T^; 12. *R. insidiosa* LMG 21421^T^. Data of strains marked with ^*^ were collected from Lu et al. (2021). Symbols: +, positive; −, negative; −/+, weak activity.

In the Gen III assays, all tested strains were positive or weakly positive for the utilization of the carbon sources α-D-glucose, D-fructose, D-galactose, D-fructose-6-PO_4_, L-aspartic acid, L-glutamic acid, L-histidine, β-hydroxy-D, L-butyric acid, acetoacetic acid, and acetic acid; were resistant to the inhibitory chemicals 1% NaCl, rifamycin SV, lincomycin, vancomycin, tetrazolium violet, tetrazolium blue, potassium tellurite and aztreonam; could not use carbon sources such as D-maltose, sucrose, stachyose, D-raffinose, α-D-lactose, β-methyl-D-glucoside, N-acetyl-D-galactosamine, N-acetyl neuraminic acid, inosine, D-sorbitol, Myo-inositol, D-serine, gelatin, glycyl-L-proline, and D-malic acid; and were unable to grow in the presence of several sensitive chemicals including 4 and 8% NaCl, minocycline, lithium chloride, sodium butyrate, and sodium bromate. In addition, the eight isolated strains shared several phenotypic features, such as being positive for growth under growth conditions of pH 6 or supplementation with N-acetyl-D-glucosamine, D-fucose, L-fucose, 1% sodium lactate, niaproof 4, L-lactic acid, α-keto-glutaric acid, L-malic acid, nalidixic acid, and tween 40. In addition, they could not use D-turanose and pectin as substrates. However, strain 21MJYT02-11^T^ had unique features, such as the utilization of L-arginine and D-serine, and the inability to use L-galactonic acid lactone (Table 2).

### Chemotaxonomic profiles

The major polar lipids present in strains of 21YRMH01-3^T^, 21MJYT02-10^T^, and 21MJYT02-11^T^ were phosphatidylethanolamine, diphosphatidylglycerol, phosphatidylglycerol, and an unidentified aminophospholipid; however, strain 21YRMH01-3^T^ contained more lipids than the other strains. In addition to the main polar lipids, one phosphatidylglycerol and several unidentified lipids, including three aminolipids, two aminophospholipids, a glycolipid, two lipids, and four phospholipids, were also present in strain 21YRMH01-3^T^ (Supplementary Figure S4).

Isoprenoid quinone analysis showed that ubiquinones Q-7 and Q-8 were present in all tested strains, and the latter was the predominant quinone. In strain 21YRMH01-3^T^, Q-8 represented 70.73% of isoprenoid quinones, which was 78.20% for strain 21MJYT02-11^T^, and 73.90% for strain 21MJYT02-10^T^. The results differ from *R. wenshanensis* 56D2^T^, which has Q-8 as the sole respiratory quinone (Lu et al., 2022a).

The fatty acid profiles from gas chromatography analysis showed that conserved components for all *Ralstonia* strains were C_14:0_, C_16:0_, C_18:0_, summed features 2 (C_14:0_ 3-OH/C_16:1_ iso I), summed features 3 (C_16:1_ *ω*7*c* and/or C_16:1_ *ω*6*c*), and summed features 8 (C_18:1_ *ω*6*c* and/or C_18:1_ *ω*7*c*), as shown in Table 3. In addition, the primary fatty acids (>10% of the total fatty acids) of strain 21YRMH01-3^T^ were identified as summed features 3 (C_16:1_ *ω*7*c* and/or C_16:1_ *ω*6*c*), summed features 8 (C_18:1_ *ω*7*c* and/or C_18:1_ *ω*6*c*), and saturated fatty acid C_16:0_, which were the same as those of strains 21MJYT02-10^T^, 21LDWP02-16, 22TCCZM01-4, 22TCJT01-1, 22TCJT01-2, 22TCCZM03-6 and the four reference strains. However, they were different from strain 21MJYT02-11^T^, whose primary fatty acids were identified as C_16:0_, summed features 3 (C_16:1_ *ω*7*c* and/or C_16:1_ *ω*6*c*) and cyclo-C_17:0_. Notably, saturated cyclo-C_17:0_ was absent in strain 21YRMH01-3^T^, but present in the other tested strains, and hydroxy C_16:1_ 2-OH was not identified in strain 21MJYT02-11^T^, but was present in all other analyzed strains. The data further supported the separate species status of strains 21YRMH01-3^T^ and 21MJYT02-11^T^.

**Table 3:** Cellular fatty acid profiles of the soil strains isolated in this study and their closely related type strains of the genus *Ralstonia*.

Fatty acids	1	2	3	4	5	6	7	8	9	10	11	12
Saturated												
C_14:0_	4.02 ± 0.38	4.66 ± 0.10	4.84 ± 0.25	5.60 ± 0.20	6.05 ± 0.33	4.91 ± 0.17	4.67 ± 0.08	5.28 ± 0.03	5.71 ± 0.65	4.67 ± 0.46	4.25 ± 0.64	4.87 ± 0.50
C_16:0_	25.50 ± 0.55	28.47 ± 0.38	27.27 ± 1.01	24.63 ± 2.30	23.59 ± 1.19	32.58 ± 0.31	34.02 ± 0.15	28.26 ± 1.36	29.94 ± 4.30	33.55 ± 2.75	30.34 ± 3.84	28.82 ± 3.97
C_18:0_	3.12 ± 0.28	1.84 ± 0.88	4.62 ± 0.54	2.14 ± 0.27	3.66 ± 2.15	2.77 ± 0.25	2.56 ± 0.06	2.74 ± 0.79	2.03 ± 0.74	1.90 ± 1.04	2.74 ± 0.79	2.78 ± 1.44
Cyclo-C_17:0_	–	3.51 ± 0.28	2.90 ± 0.19	2.97 ± 0.65	2.94 ± 0.40	4.50 ± 0.44	19.51 ± 0.36	3.05 ± 0.13	8.70 ± 1.17	TR	3.70 ± 0.94	4.89 ± 2.03
Unsaturated												
C_18:1_ *ω*9c	–	–	–	–	–	–	–	–	2.87 ± 1.50	1.21 ± 0.58	3.03 ± 0.71	2.57 ± 0.41
Hydroxy												
C_14:0_ 2-OH	TR	1.65 ± 0.03	1.31 ± 0.10	1.47 ± 0.10	1.65 ± 0.09	1.43 ± 0.11	1.93 ± 0.10	1.75 ± 0.01	1.59 ± 0.19	1.05 ± 0.19	–	1.01 ± 0.17
C_16:1_ 2-OH	0.99 ± 0.06	1.43 ± 0.02	1.67 ± 0.18	1.45 ± 0.07	2.14 ± 0.13	1.44 ± 0.22	–	TR	TR	TR	1.81 ± 0.45	TR
C_18:1_ 2-OH	4.18 ± 2.55	1.37 ± 0.21	2.57 ± 0.06	2.80 ± 0.17	3.26 ± 0.02	–	TR	–	2.25 ± 0.35	2.26 ± 0.74	1.87 ± 0.25	2.29 ± 0.59
Summed features^*^												
2	7.01 ± 0.28	6.86 ± 0.01	7.62 ± 0.34	8.55 ± 0.79	8.35 ± 0.57	7.98 ± 0.34	6.83 ± 0.20	8.42 ± 0.34	8.24 ± 1.86	8.38 ± 1.04	8.97 ± 1.67	7.93 ± 1.92
3	30.62 ± 2.92	33.60 ± 0.16	32.14 ± 1.09	35.59 ± 2.81	36.31 ± 1.53	29.38 ± 0.68	19.67 ± 0.28	32.88 ± 0.90	23.90 ± 2.51	33.91 ± 4.17	25.37 ± 0.58	28.65 ± 1.65
8	23.13 ± 0.84	12.07 ± 0.11	14.82 ± 0.42	11.47 ± 0.88	12.51 ± 0.65	13.49 ± 0.34	7.18 ± 0.14	14.82 ± 0.48	11.42 ± 4.48	10.07 ± 5.86	10.90 ± 3.48	14.24 ± 3.94

Strains: 1. 21YRMH01-3^T^; 2. 21MJYT02-10^T^; 3. 21LDWP02-16; 4. 22TCCZM01-4; 5. 22TCJT01-1; 6. 22TCJT01-2; 7. 21MJYT02-11^T^; 8. 22TCCZM03-6; 9. *R. wenshanensis* 56D2^T^; 10. *R. pickettii* JCM 5969^T^; 11. *R. mannitolilytica* JCM 11284^T^; 12. *R. insidiosa* LMG 21421^T^. The data are expressed as percentages of the total fatty acids. Compounds at a percentage of 1.0% or higher are listed. TR, trace amount (less than 1.0% of the total); −, not detected.

^*^Summed features are fatty acids that cannot be resolved reliably from another fatty acid using the chromatographic conditions chosen. The MIDI system groups these fatty acids as one feature with a single feature 2 comprises C_14:0_ 3-OH/C_16:1_ iso I. Summed feature 3 comprises C_16:1_ *ω*7*c* and/or C_16:1_ *ω*6*c*. Summed feature 8 comprises C_18:1_ *ω*6*c* and/or C_18:1_ *ω*7*c*.

## Conclusion

Based on the above data, we concluded that strains 21YRMH01-3^T^, 21MJYT02-10^T^, and 21MJYT02-11^T^ represent three novel species in the genus *Ralstonia*, for which the names *Ralstonia chuxiongensis* sp. nov., *Ralstonia mojiangensis* sp. nov., and *Ralstonia soli* sp. nov. are proposed, respectively.

### Description of *Ralstonia chuxiongensis* sp. nov.

*Ralstonia chuxiongensis* (chu. xiong. en’sis. N.L. fem. adj. *chuxiongensis*, referring to Chuxiong city in China, where the type strain was isolated).

Cells are Gram-stain negative, aerobic, non-sporulating, motile with two polar flagella, and rod-shaped, 0.79–1.14 μm wide, and 1.70–2.86 μm long (avg. 0.95 ± 0.09 μm × 2.22 ± 0.38 μm; *n* = 20). The species grows on NA, LB, TSA, PDA, mSMSA, CPG, and TZC agars, with a better growth on the latter two media. Colonies on TSA plates after incubation for 2 days at 28°C are round, opaque, raised, and smooth-surfaced, with an entire edge and 1.5 mm in diameter. Cells grow at 10–40°C (optimum, 25°C), 0–5% NaCl (w/v; optimum, 0%), and pH 5.0–9.0 (optimum, pH 7.0). Catalase and oxidase are positive. In the API 20NE assays, the strain was positive for glucose, arabinose, mannitol, N-acetylglucosamine, potassium gluconate, capric acid, malate, and trisodium citrate assimilation; negative for nitrate reduction, indole production, glucose fermentation, arginine dihydrolase, urease, and β-galactosidase activities, gelatin hydrolysis, mannose, maltose, adipic acid, and phenylacetic acid assimilations. In the API ZYM tests, the strain was positive for alkaline phosphatase, esterase (C4), esterase lipase (C8), lipase (C14), leucine arylamidase, acid phosphatase, naphthol-AS-BI-phosphohydrolase; and negative for valine arylamidase, cystine arylamidase, trypsin, α-chymotrypsin, α-galactosidase, β-galactosidase, β-glucuronidase, α-glucosidase, β-glucosidase, N-acetyl-β-glucosaminidase, α-mannosidase, and α-fucosidase. In the Gen III analysis, the strain is positive for the use of D-fructose, D-galactose, D-fucose, L-fucose, D-fructose-6-PO_4_, L-alanine, L-aspartic acid, L-glutamic acid, L-histidine, L-pyroglutamic acid, L-serine, D-galacturonic acid, L-galactonic acid lactone, D-glucuronic acid, glucuronamide, mucic acid, quinic acid, D-saccharic acid, L-lactic acid, α-keto-glutaric acid, tween 40, β-hydroxy-D, L-butyric acid, acetoacetic acid, propionic acid, and acetic acid; and is resistant to pH 5 and 6, 1% NaCl, 1% sodium lactate, fusidic acid, troleandomycin, rifamycin SV, lincomycin, guanidine HCl, niaproof 4, vancomycin, tetrazolium violet, nalidixic acid, tetrazolium blue, potassium tellurite and aztreonam. The major polar lipids of strain 21YRMH01-3^T^ are diphosphatidylglycerol, phosphatidylethanolamine, and an unidentified aminophospholipid. The bacterium has two ubiquinones, Q-7 and Q-8, with the latter being predominant. The major fatty acids are summed features 3 (C_16:1_ *ω*7*c* and/or C_16:1_ *ω*6*c*), summed features 8 (C_18:1_ *ω*7*c* and/or C_18:1_ *ω*6*c*) and C_16:0_.

The type strain is 21YRMH01-3^T^ (=GDMCC 1.3534^T^ = JCM 35818^T^), isolated from tobacco-planting soil in 2021 in Chuxiong, Yunnan, PR China. The GenBank accession number for the 16S rRNA gene sequence of the type strain is ON844322.1. The draft genome sequence was submitted to DDBJ/ENA/GenBank under the Bioproject number PRJNA839150 with the accession JAMYWC000000000. The genomic DNA G + C content of the type strain is 63.5%.

### Description of *Ralstonia mojiangensis* sp. nov.

*Ralstonia mojiangensis* (mo. jiang. en’sis. N.L. fem. adj. *mojiangensis*, referring to Mojiang town in China, where the type strain was isolated).

Cells are Gram-stain negative, aerobic, non-sporulating, motile with two polar flagella, rod-shaped, 0.77–0.94 μm wide, and 1.92–3.25 μm long (avg. 0.89 ± 0.05 μm × 2.53 ± 0.40 μm; *n* = 20). The species grows on LB, TSA, PDA, mSMSA, TZC, CPG, and NA agars, and most strains grow better on the latter two media. Colonies on TSA plates after incubation for 2 days at 28°C are round, raised, with glistening surfaces with entire edges, and 1.2 mm in diameter. Cells grow at 10–40°C (optimum, 25°C), 0–5% NaCl (w/v; optimum, 0%), and pH 5.0–9.0 (optimum, pH 6.0–7.0). Both catalase and oxidase are positive. In the API 20NE assays, the strain was positive for the hydrolysis of esculin; the assimilation of glucose, arabinose, mannitol, N-acetylglucosamine, potassium gluconate, malate, and trisodium citrate; and negative for the production of indole; the fermentation of glucose; the hydrolysis of gelatin; the enzymatic activities of arginine dihydrolase, urease, and β-galactosidase; and the assimilation of mannose, maltose, adipic acid, and phenylacetic acid. In the API ZYM tests, the strain was positive for the enzymatic activities of esterase (C4), esterase lipase (C8), lipase (C14), and leucine arylamidase; negative for alkaline phosphatase, cystine arylamidase, trypsin, α-chymotrypsin, acid phosphatase, α-galactosidase, β-galactosidase, β-glucuronidase, α-glucosidase, β-glucosidase, N-acetyl-β-glucosaminidase, α-mannosidase, and α-fucosidase. In the Gen III analysis, the strain could use D-fructose- 6-PO_4_, L-glutamic acid, D-galacturonic acid, L-galactonic acid lactone, D-glucuronic acid, glucuronamide, α-keto-glutaric acid and tween 40 as carbon sources, but were unable to utilize carbon sources such as D-maltose, sucrose, D-turanose, stachyose, D-raffinose, α-D-lactose, β-methyl-D-glucoside, N-acetyl-D-galactosamine, N-acetyl neuraminic acid, D-mannose, inosine, D-sorbitol, Myo-inositol, D-serine, gelatin, glycyl-L-proline, L-arginine, pectin, D-malic acid, and α-hydroxybutyric acid. The strain is resistant to inhibitory chemicals such as 1% NaCl, 1% sodium lactate, rifamycin SV, lincomycin, niaproof 4, vancomycin, tetrazolium violet, tetrazolium blue and nalidixic acid, and sensitive to 4 and 8% NaCl, minocycline, lithium chloride, sodium butyrate, and sodium bromate.

The bacterium has two ubiquinones, Q-7 and Q-8, with the latter being predominant. The predominant fatty acids are summed features 3 (C_16:1_ *ω*7*c* and/or C_16:1_ *ω*6*c*), summed features 8 (C_18:1_ *ω*7*c* and/or C_18:1_ *ω*6*c*) and C_16:0_.

The type strain is 21MJYT02-10^T^ (=GDMCC 1.3531^T^ = JCM 35816^T^), isolated from tobacco-planting soil in 2021 from Mojiang town, Puer, Yunnan, PR China. The GenBank accession number for the 16S rRNA gene sequence of the type strain is ON797091.1. The genome sequence was submitted to DDBJ/ENA/GenBank under the Bioproject number PRJNA839150 with the accession JAMXHU000000000. The genomic DNA G + C content of the type strain is 63.6%.

### Description of *Ralstonia soli* sp. nov.

*Ralstonia soli* (so’li. L. gen. neut. n. *soli*, of soil, the source of the type strain).

Cells are Gram-stain negative, aerobic, non-sporulating, motile with two polar flagella, and rod-shaped, 0.81–1.00 μm wide, and 1.85–2.84 μm long (avg. 0.90 ± 0.06 μm × 2.22 ± 0.33 μm; *n* = 13). The strain grows on LB, NA, PDA, TSA, mSMSA, CPG, and TZC agars. Colonies on TSA plates after incubation for 2 days at 28°C are round, opaque, raised, with glistening surfaces with entire edges and 1.5 mm in diameter. Cells grow at 10–40°C (optimum, 25°C), 0–5% NaCl (w/v; optimum, 0%), and pH 5.0–10.0 (optimum, pH 7.0). Catalase and oxidase are positive. In the API 20NE assays, the strain was positive for the hydrolysis of esculin; the assimilation of glucose, arabinose, mannitol, N-acetylglucosamine, potassium gluconate, capric acid, malate, and trisodium citrate; and negative for the reduction of nitrates; the production of indole; the fermentation of glucose; the hydrolysis of gelatin; for arginine dihydrolase, urease, and β-galactosidase activities; and the assimilation of mannose, maltose, adipic acid, and phenylacetic acid. The API ZYM test results showed that the strain is positive for enzymatic reactions of alkaline phosphatase, esterase (C4), esterase lipase (C8), lipase (C14), leucine arylamidase, acid phosphatase, and naphthol-AS-BI-phosphohydrolase; and negative for valine arylamidase, cystine arylamidase, trypsin, α-chymotrypsin, α-galactosidase, β-galactosidase, β-glucuronidase, α-glucosidase, β-glucosidase, N-acetyl-β-glucosaminidase, α-mannosidase, and α-fucosidase. In the Gen III analysis, the strain could utilize L-glutamic acid, L-histidine, L-pyroglutamic acid, L-serine, D-galacturonic acid, L-galactonic acid lactone, D-glucuronic acid, glucuronamide, mucic acid, quinic acid, D-saccharic acid, L-lactic acid, α-keto-glutaric acid, tween 40, β-hydroxy-D, L-butyric acid, acetoacetic acid, propionic acid and acetic acid as carbon sources, and was resistant to pH 5 and 6 conditions and inhibitory chemicals such as 1% NaCl, 1% sodium lactate, fusidic acid, troleandomycin, rifamycin SV, lincomycin, guanidine HCl, niaproof 4, vancomycin, tetrazolium violet, tetrazolium blue, nalidixic acid, potassium tellurite, and aztreonam. The major polar lipids of strain 21MJYT02-11^T^ are phosphatidylethanolamine, diphosphatidylglycerol, and two unidentified aminophospholipids. The bacterium has two ubiquinones, Q-7 and Q-8, with the latter being predominant. The predominant fatty acids are C_16:0_, summed features 3 (C_16:1_ *ω*7*c* and/or C_16:1_ *ω*6*c*) and cyclo-C_17:0_.

The type strain is 21MJYT02-11^T^ (=GDMCC 1.3532^T^ = JCM 35817^T^), isolated in 2021 from bulk soil sampled from Mojiang town, Puer, Yunnan, PR China. The GenBank accession number for the 16S rRNA gene sequence of the type strain is ON797092.1. The genome sequence was submitted to DDBJ/ENA/GenBank under the Bioproject number PRJNA839150 with the accession JAMXHT000000000. The genomic DNA G + C content of the type strain is 64.1%.

## Data availability statement

The datasets presented in this study can be found in online repositories. The names of the repository/repositories and accession number(s) can be found in the article/Supplementary material.

## Author contributions

C-HL, Z-LL, YJ, and Z-YX designed the research and project outline. XS, Z-MZ, W-LL, XH, Y-XX, and LG collected the soil sample. C-HL, WC, S-YZ, Q-JW, NJ, and X-TG performed the deposition and polyphasic taxonomy. C-HL, Y-YZ, and J-YL conducted the genome analysis. C-HL, L-QZ, YJ, and Z-YX drafted the manuscript. All authors read and approved the final manuscript.

## Funding

This research was supported by the Yunnan Provincial Tobacco Monopoly Bureau (grants 2023530000241014, 2020530000241013, 2018530000241006, and 2019530000241007), the National Natural Science Foundation of China (32260702), the Yunnan Fundamental Research Projects (202301AT070508), and the China National Tobacco Corporation [110202201019(LS-03)].

## Conflict of interest

XS and LG are employed by the Puer Branch of Yunnan Tobacco Company, Z-MZ and Y-XX are employed by the Baoshan Branch of Yunnan Tobacco Company, W-LL is employed by the Zhaotong Branch of Yunnan Tobacco Company, XH is employed by the Chuxiong Branch of Yunnan Tobacco Company, and Z-LL is employed by the China National Tobacco Corporation Yunnan Company, respectively.

The remaining authors declare that the research was conducted in the absence of any commercial or financial relationships that could be construed as a potential conflict of interest.

## Publisher’s note

All claims expressed in this article are solely those of the authors and do not necessarily represent those of their affiliated organizations, or those of the publisher, the editors and the reviewers. Any product that may be evaluated in this article, or claim that may be made by its manufacturer, is not guaranteed or endorsed by the publisher.

## Footnotes

^1^https://lpsn.dsmz.de/

^2^www.snapgene.com

^3^http://tree.bio.ed.ac.uk/software/figtree/

## Abbreviations

ANI: average nucleotide identity
ARO: antibiotic resistance gene ontology
CPG: casamino acid peptone glucose
*egl*: endoglucanase
GBDP: Genome BLAST Distance Phylogeny approach
GTDB: genome taxonomy database
dDDH: digital DNA–DNA hybridization
JCM: Japan Collection of Microorganisms
LA: Luria-Bertani agar
LB: Luria-Bertani
ML: maximum-likelihood
MP: maximum-parsimony
mSMSA: modified selective medium South Africa
NJ: neighbor-joining
OGRI: overall genome-related indexes
PDA: potato dextrose agar
SMM: modified semi-solid motility medium
TSA: trypticase soy agar
TYGS: Type (strain) Genome Server
TZC: Kelman’s tetrazolium chloride
