# Supplementary material for: Ralstonia chuxiongensis sp. nov., Ralstonia mojiangensis sp. nov., and Ralstonia soli sp. nov., isolated from tobacco fields, are three novel species in the family Burkholderiaceae
Source: Front Microbiol. 2023 May 5;14:1179087. doi: 10.3389/fmicb.2023.1179087 (PMC10196183; doi:10.3389/fmicb.2023.1179087)
Supplement: Supplementary file 1 [file Data_Sheet_1.docx]

**Supplementary information**

**Supplementary Figure 1** A neighbor-joining phylogenetic tree based on 16S rRNA sequences (1397 bp) shows the relationship between soil strains and other type species in the genus *Ralstonia*. The tree was constructed using MEGA 11.0. Bootstrap values (>50) are shown at branch nodes as percentages of 1000 replications. ^T^ indicates the type strain. Sequences from soil strains obtained in this study are indicated in bold. *Cupriavidus necator* N-1^T^ is used as an outgroup. The NCBI accession numbers are shown in parentheses. Bar, 0.005 substitutions per nucleotide position.

**Supplementary Figure 2** Maximum-parsimony phylogenetic tree based on 16S rRNA sequences (1397 bp) showing the relationship between soli strains and their closely related species. The tree was constructed using MEGA 11.0. Bootstrap values (≥50) are shown at branching points as percentages of 1000 replicates. ^T^ indicates the type strain. Sequences from soil strains isolated in this study are highlighted in bold. Gene accession numbers are shown in parentheses. *Cupriavidus necator* N-1^T^ is used as an outgroup.

**Supplementary Figure 3** The phylogenomic tree shows the relationship between soil strains obtained in this study and their closely related type strains of the genus *Ralstonia*. Evolutionary analyses were generated using the Type (Strain) Genome Server (Meier-Kolthoff and Göker, 2019). The numbers above branches are Genome BLAST Distance Phylogeny approach (GBDP) pseudo-bootstrap support values > 50% from 100 replicates. The tree is rooted at the midpoint. ^T^ indicates the type strain. Soil strains isolated in this study are highlighted in bold. *Cupriavidus necator* N-1^T^ is used as an outgroup. Genomic accession numbers are shown in parentheses. The scale bar equals 0.02 changes per nucleotide position.

**
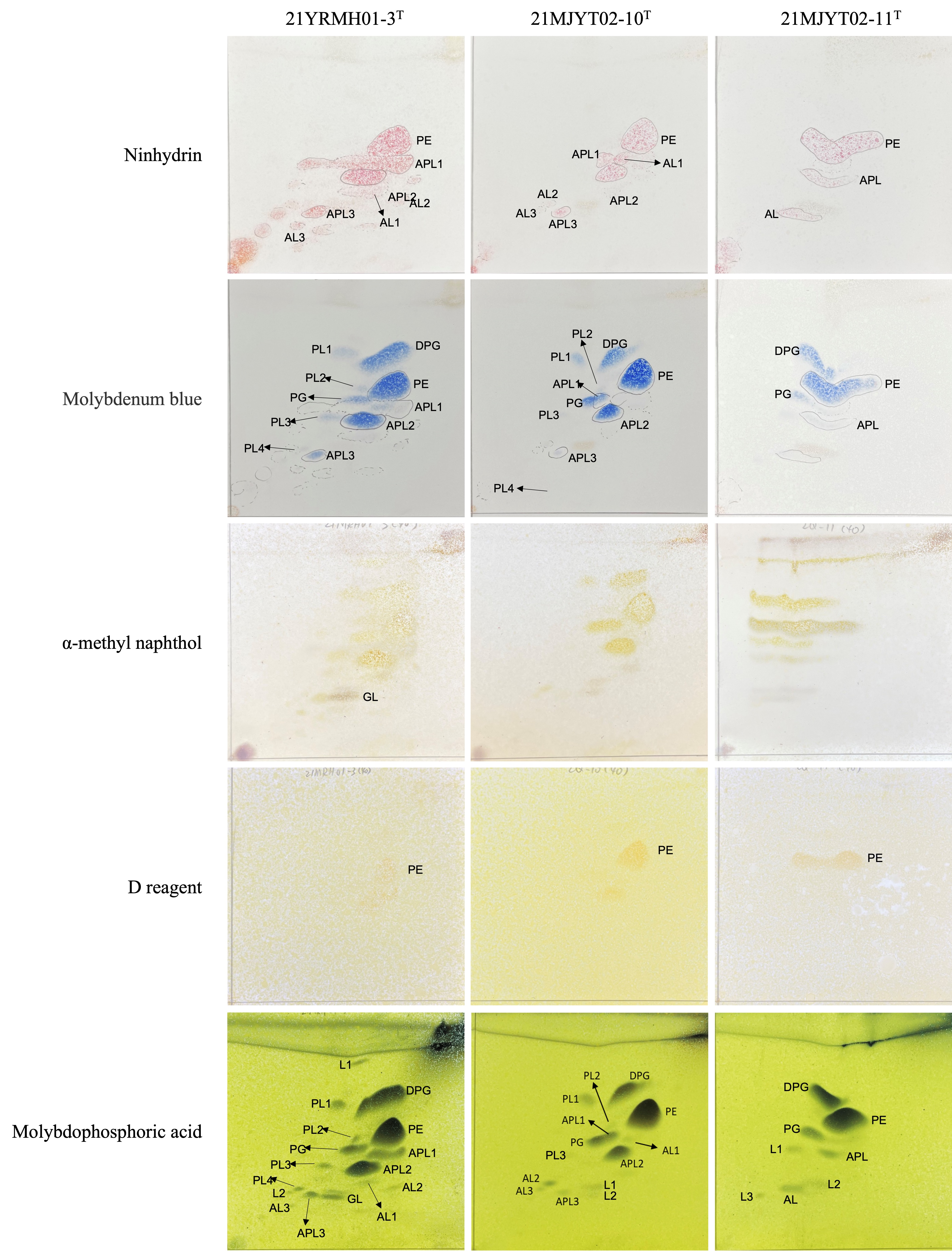
**

**Supplementary Figure 4** Two-dimensional TLC plates of polar lipids were extracted from three soil strains obtained in this study. Left images for strain 21YRMH01-3^T^, central images for strain 21MJYT02-10^T^, and right images for strain 21MJYT02-11^T^, respectively. Reagents, including ninhydrin, molybdenum blue, α-methyl naphthol and D reagent, were sprayed on the plates to identify lipid functional groups. The plates were sprayed with 10% (v/v) molybdophosphoric acid to show all polar lipids. AL stands for unidentified aminolipid; APL stands for unidentified aminophospholipid; DPG stands for diphosphatidylglycerol; GL stands for unidentified glycolipid; L stands for unidentified lipid; PE stands for phosphatidylethanolamine; PG stands for phosphatidylglycerol; PL stands for unidentified phospholipid.

**Supplementary Table 1** Strain list used in this study

| Species/Subspecies | Strain | Source | Country | Accession No. | 16S rRNA | Reference |
| --- | --- | --- | --- | --- | --- | --- |
| *Ralstonia chuxiongensis* | 21YRMH01-3^T^ (=GDMCC 1.3534^T^= JCM 35818^T^) | Soil | China | JAMYWC000000000.1 | ON844322.1 | This study |
|  | 26 | Soil | USA | JYOB01000000.1 | - | (Roco et al., 2017) |
| *R. mojiangensis* | 21MJYT02-10^T^ (=GDMCC 1.3531^T^= JCM 35816^T^) | Soil | China | JAMXHU000000000.1 | ON797091.1 | This study |
|  | 21LDWP02-16 | Soil | China | JAOCQK000000000.1 | OP404236.1 | This study |
|  | 22TCCZM01-4 | Soil | China | JAOCQJ000000000.1 | OP404237.1 | This study |
|  | 22TCJT01-1 | Soil | China | JAOCQI000000000.1 | OP404238.1 | This study |
|  | 22TCJT01-2 | Soil | China | JAOCQH000000000.1 | OP404239.1 | This study |
| *R. soli* | 21MJYT02-11^T^ (=GDMCC 1.3532^T^= JCM 35817^T^) | Soil | China | JAMXHT000000000.1 | ON797092.1 | This study |
| *R. wenshanensis* | 56D2^T^(=CCTCC AB 2021466^T^= GDMCC 1.2886^T^=JCM 35178^T^) | Soil | China | CP076413.1, CP076412.1 | MZ399217.1 | (Lu et al., 2022) |
|  | 22TCCZM03-6 | Soil | China | JAOCQG000000000.1 | OP404240.1 | This study |
| *R. pickettii* | K-288^T^(=JCM 5969^T^=ATCC 27511^T^=CCUG 3318^T^=CFBP 2459^T^=CIP 73.23^T^=DSM 6297 ^T^=HAMBI 2158^T^= LMG 5942^T^=NBRC 102503^T^= NCTC 11149^T^) | Human | USA | CP066771.1, CP066772.2, CP066773.1 | LN681565.1 | (Yabuuchi et al., 1995; Daligault et al., 2014) |
| *R. mannitolilytica* | LMG 6866^T^(=JCM 11284^T^= CCUG 38408^T^=CCUG 45027 ^T^= DSM 17512^T^=NCIB 10805^T^=NCIMB 10805^T^) | Human blood | England | CAJPVG000000000.1 | AJ270258.1 | (De Baere et al., 2001) |
| *R. insidiosa* | LMG 21421^T^(=CCUG 46789^T^=DSM 17714^T^= AU2944^T^） | Human sputum | USA | VZPV00000000.1 | AF488779.1 | (Coenye et al., 2003) |
| *R. solanacearum* | LMG 2299^T^(=A. Kelman 60-1^T^=ATCC 11696^T^=CCUG 14272^T^= CFBP 2047^T^=CIP 104762^T^=DSM 9544^T^=ICMP 5712^T^=JCM 10489^T^=K60-1^T^=NCAIM B.01459^T^=NCPPB 325^T^=NRRL B-3212^T^) | Tomato | USA | NCTK00000000.1 | EF016361.1 | (Yabuuchi et al., 1995; Remenant et al., 2012) |
| *R. pseudosolanacearum* | LMG 9673^T^(= NCPPB 10229^T^=NCPPB 1029^T^=UQRS 461^T^) | *Pelargonium capitatum* | Africa | CP103851.1, CP103852.1 | CP103852.1 | (Safni et al., 2014), This study |
| *R. syzygii* | LLRS-1 | Tobacco | China | CP046729.1, CP046730.1 | - | (Lu et al., 2021) |
| *R. syzygii* subsp*. celebesensis* | LMG 27706^T^(=DSM 27477^T^= R-46908^T^= UQRS 627^T^) | Banana | Indonesia | N/A | KC757073.1 | (Remenant et al., 2010; Safni et al., 2014) |
| *R. syzygii* subsp*. celebesensis* | UGMSS_Db01 | Banana | Indonesia | CP068285.1, CP068286.1 | CP068285.1 | (Prakoso et al., 2022) |
|  | R 229 (=ICMP 10001= T 389= LMG 27886) | Banana | Indonesia | FR854066.1- FR854085.1 | N/A | (Remenant et al., 2011; Safni et al., 2014) |
| *R. syzygii* subsp*. indonesiensis* | LMG 27703^T^(=DSM 27478^T^= PSI 7^T^= R-46900^T^= UQRS 464^T^) | Tomato | Indonesia | FP885906.2, FP885891.2 | KC757057.1 | (Remenant et al., 2010; Safni et al., 2014) |
| *R. syzygii* subsp*. syzygii* | LMG 10661^T^(=ATCC 49543^T^=CCUG 32781^T^=DSM 7385^T^=ICMP 10915^T^= NCPPB 3446^T^=R 001^T^) | Clove | Indonesia | CAKKOY000000000.1 | U28237.1 | (Remenant et al., 2011; Safni et al., 2014) |
| *Cupriavidus necator* | N-1^T^(=ATCC 43291^T^=CCUG 52238^T^=CIP 103161^T^=DSM 13513^T^=LMG 8453^T^) | Soil | USA | CP002877.1-CP002880.1 | NR_102851.1 | (Poehlein et al., 2011) |

**Supplementary Table 2** 16S rDNA sequence similarities between four type strains of novel species and their closely related species within the genus Ralstonia

| Strain | 21YRMH01-3^T^ | 21MJYT02-10^T^ | 21MJYT02-11^T^ | *R. wenshanensis* 56D2^T^ |
| --- | --- | --- | --- | --- |
| *R. chuxiong* 21YRMH01-3^T^ | 100.00 | 99.86 | 98.34 | 98.70 |
| *R. mojiangensis* 21MJYT02-10^T^ | 99.86 | 100.00 | 98.49 | 98.85 |
| *R. mojiangensis* 22TCCZM01-4 | 99.86 | 100.00 | 98.49 | 98.85 |
| *R. mojiangensis* 22TCJT01-2 | 99.86 | 100.00 | 98.49 | 98.85 |
| *R. mojiangensis* 21LDWP02-16 | 99.78 | 99.93 | 98.42 | 98.78 |
| *R. mojiangensis* 22TCJT01-1 | 99.78 | 99.93 | 98.42 | 98.78 |
| *R. pickettii* K-288^T^ | 99.71 | 99.86 | 98.34 | 98.70 |
| *R. wenshanensis* 56D2^T^ | 98.70 | 98.85 | 99.64 | 100.00 |
| *R. wenshanensis* 22TCCZM03-6 | 98.70 | 98.85 | 99.64 | 100.00 |
| *R. insidiosa* LMG 21421^T^ | 98.42 | 98.56 | 97.34 | 97.41 |
| *R. mannitolilytica* LMG 6866^T^ | 98.35 | 98.49 | 98.49 | 98.42 |
| *R. soli* 21MJYT02-11^T^ | 98.34 | 98.49 | 100.00 | 99.64 |
| *R. syzygii* subsp. *indonesiensis* PSI 07^T^ | 97.27 | 97.41 | 97.98 | 98.05 |
| *R. syzygii* subsp. *celebesensis* QRS 627^T^ | 97.19 | 97.34 | 97.76 | 97.84 |
| *R. pseudosolanacearum* LMG 9673^T^ | 97.12 | 97.27 | 97.84 | 97.91 |
| *R. syzygii* subsp. *syzygii* R001^T^ | 96.97 | 97.11 | 97.61 | 97.69 |
| *R. solanacearum* LMG 2299^T^ | 96.76 | 96.91 | 97.62 | 97.55 |
| *Cupriavidus necator* N-1^T^ | 95.89 | 96.03 | 96.17 | 96.38 |

**Supplementary Table 3** Genomic feature comparisons between eight isolated strains and their closely related strains in the genus Ralstonia

| Strain | Assembly level | Genome size (Mb) | Number of Contigs | G+C content (%) | Total genes | Protein-conding genes | RNA genes |
| --- | --- | --- | --- | --- | --- | --- | --- |
| *Ralstonia chuxiongensis* 21YRMH01-3^T^ | Scaffold | 5.61 | 22 | 63.5 | 5274 | 5191 | 56 |
| *R. chuxiongensis* 26 | Scaffold | 5.70 | 62 | 63.4 | 5253 | 5124 | 55 |
| *R. mojiangensis* 21MJYT02-10^T^ | Scaffold | 5.60 | 15 | 63.6 | 5212 | 5124 | 56 |
| *R. mojiangensis* 22TCJT01-1 | Scaffold | 5.56 | 9 | 63.5 | 5134 | 5054 | 56 |
| *R. mojiangensis* 22TCJT01-2 | Scaffold | 5.62 | 20 | 63.5 | 5139 | 5056 | 56 |
| *R. mojiangensis* 21LDWP02-16 | Scaffold | 5.45 | 14 | 63.7 | 5031 | 4952 | 51 |
| *R. mojiangensis* 22TCCZM01-4 | Scaffold | 5.51 | 17 | 63.7 | 5124 | 5038 | 56 |
| *R. wenshanensis* 56D2^T^ | Complete | 5.31 | 2 | 63.7 | 4916 | 4815 | 65 |
| *R. wenshanensis* 22TCCZM03-6 | Scaffold | 5.17 | 20 | 63.9 | 4797 | 4712 | 55 |
| *R. insidiosa* LMG 21421^T^ | Scaffold | 5.72 | 15 | 63.7 | 5362 | 5253 | 57 |
| *R. pickettii* K-288^T^ | Complete | 4.83 | 3 | 63.9 | 4532 | 4403 | 63 |
| *R. soli* 21MJYT02-11^T^ | Scaffold | 5.72 | 34 | 64.1 | 5314 | 5189 | 57 |
| *R. mannitolilytica* LMG 6866^T^ | Contig | 4.82 | 36 | 65.8 | 4546 | 4411 | 54 |
| *R. syzygii* LLRS-1 | Complete | 5.69 | 2 | 66.3 | 5059 | 4864 | 70 |
| *R. syzygii* subsp. *indonesiensis* LMG 27703^T^ | Complete | 4.00 | 2 | 66.3 | 4939 | 4746 | 68 |
| *R. syzygii* subsp. *celebesensis* UGMSS_Db01 | Complete | 5.61 | 2 | 66.5 | 4786 | 4361 | 68 |
| *R. syzygii* subsp. *syzygii* LMG 10661^T^ | Contig | 5.20 | 348 | 66.5 | 3953 | 3424 | 59 |
| *R. solonacearum* K60-1^T^ | Complete | 5.77 | 2 | 66.4 | 5216 | 4940 | 70 |
| *R. pseudosolanacearum* LMG 9673^T^ | Complete | 5.69 | 2 | 66.6 | 5129 | 4859 | 74 |
| *Cupriavidus necator* N-1^T^ | Complete | 8.48 | 4 | 65.5 | 7937 | 7508 | 88 |

**Supplementary Table 4** Secondary metabolic biosynthetic gene clusters within four soil Ralstonia strains were predicted using the bacterial antiSMASH database (Blin et al., 2021).

| Strain | Accession No. | From | To | Size (kb) | Type | Most similar known cluster | Similarity |
| --- | --- | --- | --- | --- | --- | --- | --- |
| *R. chuxiongensis* 21YRMH01-3^T^ | JAMYWC010000001.1 | 276,443 | 287,312 | 10.87 | RiPP-like | - | - |
|  | JAMYWC010000002.1 | 500,264 | 525,290 | 25.03 | betalactone | - | - |
|  | JAMYWC010000002.1 | 568,720 | 580,687 | 11.97 | siderophore | - | - |
|  | JAMYWC010000002.1 | 665,913 | 707,130 | 41.22 | arylpolyene | - | - |
|  | JAMYWC010000004.1 | 290,001 | 312,138 | 22.14 | redox-cofactor | - | - |
|  | JAMYWC010000005.1 | 176,705 | 197,538 | 20.83 | terpene | - | - |
|  | JAMYWC010000007.1 | 20,782 | 46,282 | 25.50 | betalactone | - | - |
| *R. insidiosa* CCUG 46789^T^ | VZPV01000001.1 | 745,129 | 770,141 | 25.01 | betalactone | - | - |
|  | VZPV01000001.1 | 934,302 | 975,534 | 41.23 | arylpolyene | - | - |
|  | VZPV01000001.1 | 2,040,564 | 2,061,397 | 20.83 | terpene | - | - |
|  | VZPV01000001.1 | 2,072,745 | 2,084,631 | 11.89 | siderophore | putrebactin / avaroferrin | 30% |
|  | VZPV01000003.1 | 291,430 | 313,549 | 22.12 | redox-cofactor | lankacidin C | 13% |
|  | VZPV01000005.1 | 104,304 | 115,170 | 10.87 | RiPP-like | - | - |
| *R. mannitolilytica* LMG 6866^T^ | CAJPVG010000001.1 | 879,438 | 924,308 | 44.87 | arylpolyene | APE Vf | 35% |
|  | CAJPVG010000013.1 | 87,427 | 98,296 | 10.87 | RiPP-like | - | - |
|  | CAJPVG010000003.1 | 69,421 | 90,293 | 20.87 | terpene | - | - |
|  | CAJPVG010000003.1 | 94,254 | 106,134 | 11.88 | siderophore | putrebactin / avaroferrin | 30% |
|  | CAJPVG010000004.1 | 65,843 | 87,980 | 22.14 | redox-cofactor | lankacidin C | 13% |
|  | CAJPVG010000005.1 | 191,115 | 232,371 | 41.26 | arylpolyene | APE Vf | 10% |
| *R. mojiangensis* 21MJYT02-10^T^ | JAMXHU000000001.1 | 539,958 | 562,095 | 22.14 | redox-cofactor | - | - |
|  | JAMXHU000000001.1 | 1,243,264 | 1,264,097 | 20.83 | terpene | - | - |
|  | JAMXHU000000001.1 | 1,272,752 | 1,285,259 | 12.51 | siderophore | putrebactin / avaroferrin | 30% |
|  | JAMXHU000000002.1 | 206,559 | 247,776 | 41.22 | arylpolyene | APE Vf | 10% |
|  | JAMXHU000000002.1 | 341,179 | 353,155 | 11.98 | siderophore | - | - |
|  | JAMXHU000000002.1 | 395,871 | 420,898 | 25.03 | betalactone | - | - |
|  | JAMXHU000000004.1 | 425,153 | 470,017 | 44.86 | arylpolyene | APE Vf | 35% |
|  | JAMXHU000000008.1 | 6,589 | 17,458 | 10.87 | RiPP-like | - | - |
| *R. pickettii* K-288^T^ | CP066771.1 | 125,693 | 150,720 | 25.03 | betalactone | - | - |
|  | CP066771.1 | 193,774 | 205,741 | 11.97 | siderophore | - | - |
|  | CP066771.1 | 294,479 | 335,696 | 41.22 | arylpolyene | APE Vf | 10% |
|  | CP066771.1 | 1,586,897 | 1,607,730 | 20.83 | terpene | - | - |
|  | CP066771.1 | 1,692,520 | 1,740,190 | 47.67 | T1PKS | lipopolysaccharide | 8% |
|  | CP066772.2 | 1,065,544 | 1,076,413 | 10.87 | RiPP-like | - | - |
| *R. soli* 21MJYT02-11^T^ | JAMXHT010000001.1 | 641,625 | 682,863 | 41.24 | arylpolyene | - | - |
|  | JAMXHT010000010.1 | 1 | 14,923 | 14.92 | RRE-containing | lankacidin C | 13% |
|  | JAMXHT010000014.1 | 6,363 | 17,232 | 10.87 | RiPP-like | - | - |
|  | JAMXHT010000004.1 | 106,156 | 126,998 | 20.84 | terpene | - | - |
|  | JAMXHT010000007.1 | 79,123 | 124,047 | 44.92 | arylpolyene | APE Vf | 40% |
| *R. wenshanensis* 56D2^T^ | CP076412.1 | 263,775 | 274,644 | 10.87 | RiPP-like | - | - |
|  | CP076413.1 | 121,742 | 146,769 | 25.03 | betalactone | - | - |
|  | CP076413.1 | 190,196 | 202,163 | 11.97 | siderophore | - | - |
|  | CP076413.1 | 332,980 | 374,197 | 41.22 | arylpolyene | APE Vf | 10% |
|  | CP076413.1 | 1,650,405 | 1,671,238 | 20.83 | terpene | - | - |
|  | CP076413.1 | 2,273,559 | 2,295,696 | 22.14 | redox-cofactor | - | - |

**Supplementary Table 5** Potential resistance genes of the strains 21YRMH01-3^T^, 21MJYT02-10^T^, 21MJYT02-11^T^, and type strains of the genus *Ralstonia*.

| Strain | RGI criteria | Gene | AMR gene family | Drug class | Resistance mechanism | Identity of matching region/% | Length of reference sequence/% |
| --- | --- | --- | --- | --- | --- | --- | --- |
| *Ralstonia chuxiongensis*  21YRMH01-3^T^ | Strict | adeF | resistance-nodulation-cell division (RND) antibiotic efflux pump | fluoroquinolone antibiotic, tetracycline antibiotic | antibiotic efflux | 79.75 | 11.43 |
|  | Strict | adeF | resistance-nodulation-cell division (RND) antibiotic efflux pump | fluoroquinolone antibiotic, tetracycline antibiotic | antibiotic efflux | 43.94 | 11.43 |
|  | Strict | OXA-22 | OXA beta-lactamase | carbapenem, cephalosporin, penam | antibiotic inactivation | 97.08 | 44.16 |
|  | Strict | OXA-571 | OXA beta-lactamase | carbapenem, cephalosporin, penam | antibiotic inactivation | 95.20 | 44.65 |
|  | Strict | tet(D) | major facilitator superfamily (MFS) antibiotic efflux pump | tetracycline antibiotic | antibiotic efflux | 43.64 | 30.71 |
| *R. mojiangensis*  21MJYT02-10^T^ | Strict | adeF | resistance-nodulation-cell division (RND) antibiotic efflux pump | fluoroquinolone antibiotic, tetracycline antibiotic | antibiotic efflux | 43.85 | 10.29 |
|  | Strict | adeF | resistance-nodulation-cell division (RND) antibiotic efflux pump | fluoroquinolone antibiotic, tetracycline antibiotic | antibiotic efflux | 79.75 | 10.29 |
|  | Strict | OXA-571 | OXA beta-lactamase | carbapenem, cephalosporin, penam | antibiotic inactivation | 95.94 | 40.22 |
|  | Strict | OXA-899 | OXA beta-lactamase | carbapenem, cephalosporin, penam | antibiotic inactivation | 95.99 | 39.64 |
|  | Strict | tet(D) | major facilitator superfamily (MFS) antibiotic efflux pump | tetracycline antibiotic | antibiotic efflux | 44.16 | 27.66 |
| *R. soli*  21MJYT02-11^T^ | Strict | adeF | resistance-nodulation-cell division (RND) antibiotic efflux pump | fluoroquinolone antibiotic, tetracycline antibiotic | antibiotic efflux | 42.98 | 13.60 |
|  | Strict | adeF | resistance-nodulation-cell division (RND) antibiotic efflux pump | fluoroquinolone antibiotic, tetracycline antibiotic | antibiotic efflux | 79.17 | 13.60 |
|  | Strict | OXA-569 | OXA beta-lactamase | carbapenem, cephalosporin, penam | antibiotic inactivation | 86.74 | 51.61 |
|  | Strict | OXA-571 | OXA beta-lactamase | carbapenem, cephalosporin, penam | antibiotic inactivation | 92.25 | 53.14 |
|  | Strict | vanH gene in vanA cluster | vanH, glycopeptide resistance gene cluster | glycopeptide antibiotic | antibiotic target alteration | 38.54 | 44.72 |
| *R. wenshanensis*  56D2^T^ | Strict | adeF | resistance-nodulation-cell division (RND) antibiotic efflux pump | fluoroquinolone antibiotic, tetracycline antibiotic | antibiotic efflux | 43.75 | 99.15 |
|  | Strict | adeF | resistance-nodulation-cell division (RND) antibiotic efflux pump | fluoroquinolone antibiotic, tetracycline antibiotic | antibiotic efflux | 42.65 | 99.15 |
|  | Strict | adeF | resistance-nodulation-cell division (RND) antibiotic efflux pump | fluoroquinolone antibiotic, tetracycline antibiotic | antibiotic efflux | 79.65 | 100.85 |
|  | Strict | OXA-571 | OXA beta-lactamase | carbapenem, cephalosporin, penam | antibiotic inactivation | 95.57 | 100.00 |
|  | Strict | OXA-899 | OXA beta-lactamase | carbapenem, cephalosporin, penam | antibiotic inactivation | 96.35 | 100.00 |
|  | Strict | tet(D) | major facilitator superfamily (MFS) antibiotic efflux pump | tetracycline antibiotic | antibiotic efflux | 43.04 | 99.75 |
| *R. insidiosa*  CCUG 46789^T^ | Strict | adeF | resistance-nodulation-cell division (RND) antibiotic efflux pump | fluoroquinolone antibiotic, tetracycline antibiotic | antibiotic efflux | 43.75 | 6.80 |
|  | Strict | adeF | resistance-nodulation-cell division (RND) antibiotic efflux pump | fluoroquinolone antibiotic, tetracycline antibiotic | antibiotic efflux | 79.85 | 6.80 |
|  | Strict | OXA-573 | OXA beta-lactamase | carbapenem, cephalosporin, penam | antibiotic inactivation | 100.00 | 26.57 |
|  | Strict | OXA-574 | OXA beta-lactamase | carbapenem, cephalosporin, penam | antibiotic inactivation | 100.00 | 25.81 |
|  | Strict | qacJ | small multidrug resistance (SMR) antibiotic efflux pump | disinfecting agents and antiseptics | antibiotic efflux | 42.45 | 67.29 |
|  | Strict | tet(D) | major facilitator superfamily (MFS) antibiotic efflux pump | tetracycline antibiotic | antibiotic efflux | 43.38 | 18.27 |
| *R. mannitolilytica*  LMG 6866^T^ | Strict | adeF | resistance-nodulation-cell division (RND) antibiotic efflux pump | fluoroquinolone antibiotic, tetracycline antibiotic | antibiotic efflux | 79.27 | 14.83 |
|  | Strict | adeF | resistance-nodulation-cell division (RND) antibiotic efflux pump | fluoroquinolone antibiotic, tetracycline antibiotic | antibiotic efflux | 43.94 | 14.83 |
|  | Strict | OXA-443 | OXA beta-lactamase | carbapenem, cephalosporin, penam | antibiotic inactivation | 100.00 | 57.09 |
|  | Strict | OXA-444 | OXA beta-lactamase | carbapenem, cephalosporin, penam | antibiotic inactivation | 100.00 | 57.93 |
|  | Strict | qacJ | small multidrug resistance (SMR) antibiotic efflux pump | disinfecting agents and antiseptics | antibiotic efflux | 42.45 | 146.73 |
| *R. pickettii*  K-288^T^ | Strict | adeF | resistance-nodulation-cell division (RND) antibiotic efflux pump | fluoroquinolone antibiotic, tetracycline antibiotic | antibiotic efflux | 43.65 | 7.18 |
|  | Strict | adeF | resistance-nodulation-cell division (RND) antibiotic efflux pump | fluoroquinolone antibiotic, tetracycline antibiotic | antibiotic efflux | 79.37 | 7.18 |
|  | Strict | OXA-22 | OXA beta-lactamase | carbapenem, cephalosporin, penam | antibiotic inactivation | 96.70 | 27.74 |
|  | Strict | OXA-60 | OXA beta-lactamase | carbapenem, cephalosporin, penam | antibiotic inactivation | 98.15 | 28.04 |
| *R. syzygii* subsp.  *indonesiensis* PSI 7^T^ | Strict | adeF | resistance-nodulation-cell division (RND) antibiotic efflux pump | fluoroquinolone antibiotic, tetracycline antibiotic | antibiotic efflux | 42.79 | 33.43 |
|  | Strict | adeF | resistance-nodulation-cell division (RND) antibiotic efflux pump | fluoroquinolone antibiotic, tetracycline antibiotic | antibiotic efflux | 42.62 | 33.43 |
|  | Strict | adeF | resistance-nodulation-cell division (RND) antibiotic efflux pump | fluoroquinolone antibiotic, tetracycline antibiotic | antibiotic efflux | 79.41 | 33.43 |
|  | Strict | qacG | small multidrug resistance (SMR) antibiotic efflux pump | disinfecting agents and antiseptics | antibiotic efflux | 44.23 | 330.84 |
| *R. pseudosolanacearum*  LMG 9673^T^ | Strict | adeF | resistance-nodulation-cell division (RND) antibiotic efflux pump | fluoroquinolone antibiotic, tetracycline antibiotic | antibiotic efflux | 42.98 | 20.49 |
|  | Strict | adeF | resistance-nodulation-cell division (RND) antibiotic efflux pump | fluoroquinolone antibiotic, tetracycline antibiotic | antibiotic efflux | 78.64 | 20.49 |
|  | Strict | adeF | resistance-nodulation-cell division (RND) antibiotic efflux pump | fluoroquinolone antibiotic, tetracycline antibiotic | antibiotic efflux | 42.82 | 20.49 |
| *R. solanacearum*  K60^T^ | Strict | adeF | resistance-nodulation-cell division (RND) antibiotic efflux pump | fluoroquinolone antibiotic, tetracycline antibiotic | antibiotic efflux | 42.77 | 26.35 |
|  | Strict | adeF | resistance-nodulation-cell division (RND) antibiotic efflux pump | fluoroquinolone antibiotic, tetracycline antibiotic | antibiotic efflux | 42.50 | 26.35 |
|  | Strict | adeF | resistance-nodulation-cell division (RND) antibiotic efflux pump | fluoroquinolone antibiotic, tetracycline antibiotic | antibiotic efflux | 79.02 | 26.35 |

**Supplementary Table 6** Antibiotic susceptibility of soil bacteria of the *Ralstonia* and their closely related type species

Strains: 1. *R. chuxiongensis* 21YRMH01-3^T^*;* 2. *R. mojiangensis* 21MJTY02-10^T^; 3. *R. mojiangensis* 21LDWP02-16; 4. *R. mojiangensis* 22TCCZM01-4; 5. *R. mojiangensis* 22TCJT01-1; 6. *R. mojiangensis* 22TCJT01-2; 7. *R. soli* 21MJYT02-11^T^; 8. *R. wenshanensis* 56D2^T^; 9. *R. wenshanensis* 22TCCZM03-6; 10. *R. pickettii* JCM 5969^T^; 11. *R. insidiosa* LMG 21421^T^; 12. *R. syzygii* subsp. *indonesiensis* LMG 27703^T^; 13. *R. pseudosolanacearum* LMG 9673^T^; 14. *R. solanacearum* LMG 2299^T^. The data are expressed as the optical density of samples measured at a wavelength of 600 nm. NG, no growth.

| Antibiotics | 1 | *2* | 3 | 4 | 5 | 6 | 7 | 8 | 9 | 10 | 11 | 12 | 13 | 14 |
| --- | --- | --- | --- | --- | --- | --- | --- | --- | --- | --- | --- | --- | --- | --- |
| Kanamycin (50 μg/mL) | 3.34 | 0.20 | 0.41 | 0.83 | 0.16 | 0.61 | NG | 0.51 | 0.31 | 1.02 | 0.41 | NG | NG | NG |
| Tetracycline (15 μg/mL) | 1.04 | 0.34 | NG | 0.34 | NG | NG | NG | 0.31 | NG | NG | 0.62 | NG | NG | NG |
| Streptomycin (100 μg/mL) | 1.83 | 1.19 | 0.31 | 1.04 | 1.25 | 0.73 | NG | 1.12 | 0.54 | 0.87 | 1.41 | NG | NG | NG |
| Polymyxopeptide (10 μg/mL) | 3.78 | 4.03 | 1.59 | 4.28 | 3.80 | 4.58 | 4.49 | 3.75 | 3.82 | 1.34 | 2.15 | 2.08 | 2.57 | 2.67 |
| Cycloheximide (10 μg/mL) | 3.14 | 1.82 | 1.79 | 3.29 | 2.72 | 2.49 | 2.59 | 3.32 | 3.29 | 2.58 | 2.41 | 3.35 | 2.40 | 3.76 |
| Bacitracin (10 μg/mL) | 3.47 | 3.39 | 3.06 | 3.40 | 3.80 | 3.78 | 3.61 | 3.30 | 3.44 | 1.51 | 2.14 | 3.23 | 2.95 | 2.75 |
| Chloromycetin (30 μg/mL) | 3.98 | 2.61 | 1.32 | 2.68 | 2.69 | 4.17 | 1.53 | 2.21 | 3.05 | 0.62 | 1.72 | 1.70 | 1.51 | 2.56 |
| Rifamycin (25 μg/mL) | NG | NG | NG | NG | NG | NG | 1.46 | NG | NG | NG | 4.52 | NG | NG | NG |
| Ampicillin (100 μg/mL) | 2.64 | 3.42 | 1.34 | 4.18 | 2.85 | 2.75 | 2.27 | 2.64 | 3.00 | 1.18 | 1.50 | 0.73 | 0.08 | 0.05 |
| Gentamicin (100 μg/mL) | 3.10 | 3.39 | 1.88 | 4.08 | 3.28 | 2.66 | NG | 3.42 | 3.23 | 1.61 | 2.67 | NG | NG | NG |
| Ciprofloxacin (1 μg/mL) | NG | NG | NG | NG | NG | NG | NG | NG | NG | NG | NG | NG | NG | NG |

**REFERENCES**

Blin, K., Shaw, S., Kloosterman, A. M., Charlop-Powers, Z., van Wezel, G. P., Medema, Marnix H., et al. (2021). antiSMASH 6.0: Improving cluster detection and comparison capabilities. *Nucleic Acids Research* 49, W29-W35. doi: 10.1093/nar/gkab335.

Coenye, T., Goris, J., De Vos, P., Vandamme, P., and LiPuma, J. J. (2003). Classification of *Ralstonia pickettii*-like isolates from the environment and clinical samples as *Ralstonia insidiosa* sp. nov. *International Journal of Systematic and Evolutionary Microbiology* 53, 1075-1080. doi: 10.1099/ijs.0.02555-0.

Daligault, H. E., Davenport, K. W., Minogue, T. D., Broomall, S. M., Bruce, D. C., Chain, P. S., et al. (2014). Draft genome assembly of *Ralstonia pickettii* type strain K-288 (ATCC 27853). *Genome Announcements* 2, e00973-00914. doi: 10.1128/genomeA.00973-14.

De Baere, T., Steyaert, S., Wauters, G., Des Vos, P., Goris, J., Coenye, T., et al. (2001). Classification of *Ralstonia pickettii* biovar 3/‘*thomasii*’ strains (Pickett 1994) and of new isolates related to nosocomial recurrent meningitis as *Ralstonia mannitolytica* sp. nov. *International Journal of Systematic and Evolutionary Microbiology* 51, 547-558. doi: 10.1099/00207713-51-2-547.

Lu, C.-H., Chen, W., Yin, H.-H., Lin, Z.-L., Li, J.-Y., Ma, J.-H., et al. (2022). *Ralstonia wenshanensis* sp. nov., a novel bacterium isolated from a tobacco field in Yunnan, China. *International Journal of Systematic and Evolutionary Microbiology* 72, 005622. doi: 10.1099/ijsem.0.005622.

Lu, C.-H., Li, J.-Y., Mi, M.-G., Lin, Z.-L., Jiang, N., Gai, X.-T., et al. (2021). Complete genome sequence of *Ralstonia syzygii* subsp. *indonesiensis* strain LLRS-1, isolated from wilted tobacco in China. *Phytopathology* 111, 2392-2395. doi: 10.1094/phyto-04-21-0138-a.

Meier-Kolthoff, J. P., and Göker, M. (2019). TYGS is an automated high-throughput platform for state-of-the-art genome-based taxonomy. *Nature Communications* 10, 2182. doi: 10.1038/s41467-019-10210-3.

Poehlein, A., Kusian, B., Friedrich, B., Daniel, R., and Bowien, B. (2011). Complete genome sequence of the type strain *Cupriavidus necator* N-1. *Journal of Bacteriology* 193, 5017-5017. doi: 10.1128/JB.05660-11.

Prakoso, A. B., Joko, T., Soffan, A., Sari, J. P., Ray, J. D., Drenth, A., et al. (2022). Draft genome sequence of *Ralstonia syzygii* subsp. *celebesensis* from Indonesia, the causal agent of blood disease of banana. *Phytopathology* 0, PHYTO10210443A. doi: 10.1094/PHYTO-10-21-0443-A.

Remenant, B., Babujee, L., Lajus, A., Médigue, C., Prior, P., and Allen, C. (2012). Sequencing of K60, type strain of the major plant pathogen *Ralstonia solanacearum*. *Journal of Bacteriology* 194, 2742-2743. doi: 10.1128/JB.00249-12.

Remenant, B., Coupat-Goutaland, B., Guidot, A., Cellier, G., Wicker, E., Allen, C., et al. (2010). Genomes of three tomato pathogens within the *Ralstonia solanacearum* species complex reveal significant evolutionary divergence. *BMC Genomics* 11, 379. doi: 10.1186/1471-2164-11-379.

Remenant, B., de Cambiaire, J. C., Cellier, G., Jacobs, J. M., Mangenot, S., Barbe, V., et al. (2011). *Ralstonia syzygii*, the blood disease bacterium and some Asian *R. solanacearum* strains form a single genomic species despite divergent lifestyles. *PLoS One* 6, e24356. doi: 10.1371/journal.pone.0024356.

Roco, C. A., Bergaust, L. L., Bakken, L. R., Yavitt, J. B., and Shapleigh, J. P. (2017). Modularity of nitrogen-oxide reducing soil bacteria: linking phenotype to genotype. *Environmental Microbiology* 19, 2507-2519. doi: 10.1111/1462-2920.13250.

Safni, I., Cleenwerck, I., De Vos, P., Fegan, M., Sly, L., and Kappler, U. (2014). Polyphasic taxonomic revision of the *Ralstonia solanacearum* species complex: proposal to emend the descriptions of *Ralstonia solanacearum* and *Ralstonia syzygii* and reclassify current *R. syzygii* strains as *Ralstonia syzygii* subsp. *syzygii* subsp. nov., *R. solanacearum* phylotype IV strains as *Ralstonia syzygii* subsp. *indonesiensis* subsp. nov., banana blood disease bacterium strains as *Ralstonia* *syzygii* subsp. *celebesensis* subsp. nov. and *R. solanacearum* phylotype I and III strains as *Ralstonia pseudosolanacearum* sp. nov. *International Journal of Systematic and Evolutionary Microbiology* 64, 3087-3103. doi: 10.1099/ijs.0.066712-0.

Yabuuchi, E., Kosako, Y., Yano, I., Hotta, H., and Nishiuchi, Y. (1995). Transfer of two *Burkholderia* and an *Alcaligenes* species to *Ralstonia* gen. nov. *Microbiology and Immunology* 39, 897-904. doi: 10.1111/j.1348-0421.1995.tb03275.x.
